# A critical analysis of calcium carbonate mesocrystals

**DOI:** 10.1038/ncomms5341

**Published:** 2014-07-11

**Authors:** Yi-Yeoun Kim, Anna S. Schenk, Johannes Ihli, Alex N. Kulak, Nicola B. J. Hetherington, Chiu C. Tang, Wolfgang W. Schmahl, Erika Griesshaber, Geoffrey Hyett, Fiona C. Meldrum

**Affiliations:** 1 School of Chemistry, University of Leeds, Woodhouse Lane, Leeds LS2 9JT, UK; 2 Diamond Light Source, Harwell Science and Innovation Campus, Didcot, Oxfordshire OX11 0DE, UK; 3 Ludwig-Maximilians-Universität München, Department für Geo- und Umweltwissenschaften, Sektion Kristallographie, Theresienstrasse 41, 80333 München, Germany; 4 Department of Chemistry, University of Southampton, Highfield, Southampton SO17 1BJ, UK

## Abstract

The term mesocrystal has been widely used to describe crystals that form by oriented assembly, and that exhibit nanoparticle substructures. Using calcite crystals co-precipitated with polymers as a suitable test case, this article looks critically at the concept of mesocrystals. Here we demonstrate that the data commonly used to assign mesocrystal structure may be frequently misinterpreted, and that these calcite/polymer crystals do not have nanoparticle substructures. Although morphologies suggest the presence of nanoparticles, these are only present on the crystal surface. High surface areas are only recorded for crystals freshly removed from solution and are again attributed to a thin shell of nanoparticles on a solid calcite core. Line broadening in powder X-ray diffraction spectra is due to lattice strain only, precluding the existence of a nanoparticle sub-structure. Finally, study of the formation mechanism provides no evidence for crystalline precursor particles. A re-evaluation of existing literature on some mesocrystals may therefore be required.

## Abstract (web summary)

The term mesocrystal describes three-dimensional crystals formed by oriented assembly and that exhibit nanoparticle substructures. Here, the authors perform detailed structural analyses on synthetic calcium carbonate/polymer crystals, and show that common signatures used to assign mesocrystals may be unreliable.

## Graphical abstract

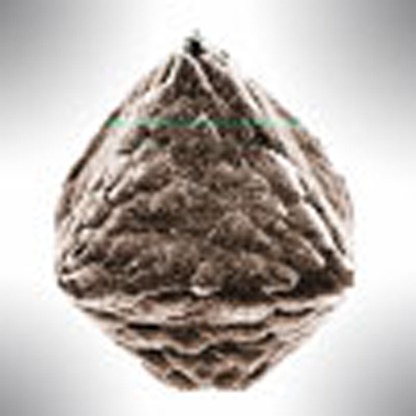

The last decade has seen enormous leaps in our understanding of solution-based crystallization processes. Together with non-classical mechanisms of nucleation^1^,^2^,^3^ and the selective entrapment of inclusions within single crystals^4^,^5^,^6^, it is now recognized that crystal growth can often occur by the aggregation of precursor units rather than by ion-by ion growth^7^,^8^,^9^. Pioneering work from Banfield *et al*.^10^, in which it was shown that single crystal TiO_2_ nanowires can form via the oriented attachment of crystalline nanoparticles, inspired much of the current research into aggregation-based crystallization. Indeed, it is now known that single crystals of many materials including TiO_2_/SnO_2_ (ref. 11), goethite^12^, PbSe^13^ and iron oxyhydroxide^14^ can form by oriented attachment, where this process operates at the nanoscale to give one-dimensional wires or irregular nanostructures^15^,^16^.

Following early demonstrations of crystal growth by oriented assembly, this concept was extended to the formation of larger, three-dimensional crystals, where these were designated as ‘mesocrystals’. The term mesocrystal was first applied to calcite and vaterite crystals (polymorphs of CaCO_3_) that were precipitated in the presence of polymer additives^17^,^18^,^19^,^20^. Mesocrystals were proposed to form via the oriented assembly of polymer-stabilized crystalline nanoparticles (Fig. 1), where evidence for this mechanism was derived from structural analysis of the product crystals. These articles created an enormous amount of interest, and >400 examples of mesocrystals have now been proposed in the literature^21^,^22^,^23^,^24^,^25^,^26^. Looking critically at this mechanism of crystallization, however, the ability to achieve perfect crystallographic register of subunits over large length scales is clearly highly challenging. The term mesocrystal has therefore recently been refined to define these particles based on their structures, rather than their formation mechanism, such that ‘a mesocrystal ideally comprises a three-dimensional array of iso-oriented single crystal particles of size 1–1,000 nm’ (ref. 27). This definition is potentially far more general and could encompass crystals that form by the assembly and subsequent crystallization of amorphous precursor particles, provided that a memory of the precursor particles is retained in the product crystal.

This evolution of ideas about mesocrystal structure and formation has resulted in a marked lack of consensus in the literature, where this problem is further exacerbated by particles sometimes being designated as mesocrystals on the basis of rather superficial structural analyses. In this article, we look critically at the subject of mesocrystals, and use calcite crystals, precipitated in the presence of polymer additives, as a test case to investigate the validity of the characterization methods commonly used. Indeed, although it is now commonplace to assign mesocrystal structures based on nanoparticulate surface structures and analyses of X-ray diffraction (XRD) data using the Scherrer equation, we demonstrate that both of these approaches may be insufficient and unsafe. We then examine the mechanism of formation of these calcite mesocrystals, and show that modifications in the crystal morphology only occur at later stages of growth. An amorphous calcium carbonate (ACC) precursor phase is also not a pre-requisite to the development of classic mesocrystal morphologies. Finally, electron backscatter diffraction (EBSD) is used to investigate the microstructures of synthetic mesocrystals, revealing a sector-like mosaic structure that contrasts markedly with the uniform nanostructure seen for a biogenic mesocrystal—a sea urchin spine^27^.

## Results

### Precipitation and characterization of calcite crystals

**Figure 2:**
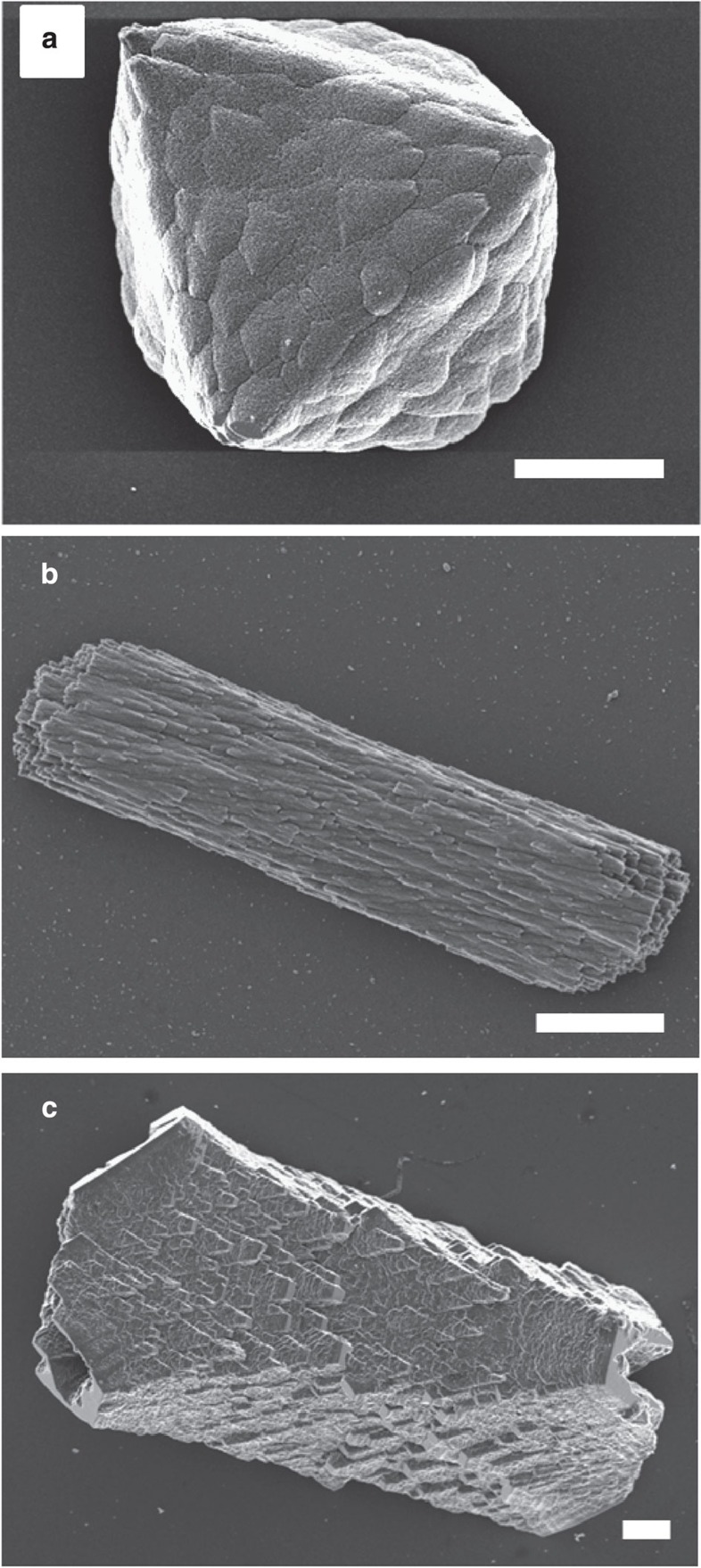
SEM images showing final calcite morphologies. (**a**) Calcite crystal precipitated in the presence of [Ca^2+^]=5 mM and [PSS-MA]=125 μg ml^−1^; (**b**) calcite crystal precipitated in the presence of [Ca^2+^]=1.5 mM and [PS-MA]= 250 μg ml^−1^; (**c**) calcite crystal precipitated in the presence of [Ca^2+^]=7 mM, [Ca^2+^]:[Co^2+^]=50:1. Scale bars, 5 μm.

A number of calcite particles that have previously been described as mesocrystals were analysed. CaCO_3_ was precipitated in the presence of the polymeric additives poly(4-styrene sulphonate-co-maleic acid) (PSS-MA) and poly(styrene-alt-maleic acid) (PS-MA; Supplementary Fig. 1), where they exhibited the anticipated morphologies of platonic/dodecahedral for PSS-MA and rod-shaped for PS-MA (Fig. 2a,b). These crystals, which are designated as calcite/polymer throughout, were then characterized using powder XRD (PXRD), small-angle X-ray scattering (SAXS), surface area analysis (based on the method by Brunauer, Emmett and Teller (BET)), scanning electron microscopy (SEM), transmission electron microscopy (TEM) and electron backscatter diffraction (EBSD), and comparison was made with control samples. These include calcite precipitated in the presence of Co^2+^ ions, which exhibits a rod-shaped form comparable to calcite/PS-MA and yet is widely accepted to be a single crystal (Fig. 2c)^28^,^29^, and also calcite crystals produced by calcite overgrowth of ≈3 μm rhombohedral calcite seeds in the presence of the selected polymers. Finally, the mechanism of formation of these crystals was investigated and was considered in light of proposed routes to calcite mesocrystals. The overgrowth sample formed part of this study and was used to investigate whether classic mesocrystal morphologies could develop on overgrowth of a rhombohedral calcite core.

### X-ray analysis of internal structures of crystals

PXRD has been widely used to prove the existence of a mesocrystal structure, where this has always been achieved using the Scherrer equation to estimate the nanoparticle size from the peak broadening. XRD peak broadening is caused by contributions from the instrument, the crystallite domain size and lattice distortion caused by microstrain^30^. Application of the Scherrer equation involves taking the peak full-width half-maximum (FWHM), removing the instrumental broadening and then assuming that all remaining broadening is caused by particle size effects alone. This has the virtue of being very straightforward and is suitable for samples where domain size (coherence length) is the major contributor to peak broadening (for example, nanoparticles). However, in most cases, ignoring strain effects is far too simplistic a model, and application of the Scherrer equation when lattice strain is present will result in an underestimate of the domain size. Full analysis of PXRD patterns using either Rietveld refinement or Williamson–Hall plots overcomes this problem and enables the individual contributions of domain size and lattice strain to the peak broadening to be determined. These approaches differ in that Rietveld refinement applies a sophisticated rigorous model and generates parameters that are refined to fit the whole pattern, while Williamson–Hall makes use of adjusted FWHM or integral breath values, separating out size and strain effects based on how they vary with the diffraction angle *θ*, and assuming that these effects are simply additive on the FWHM and integral breath. Notably, in the analyses presented here, both methods validate each other by showing the same trends (Supplementary Note 1).

A detailed synchrotron PXRD analysis of the structures of control samples (pure calcite and Co^2+^-doped calcite) and calcite/PSS-MA, seeded calcite/PSS-MA and calcite/PS-MA crystals was carried out, where the recorded patterns were modelled using a Rietveld refinement, Williamson–Hall plots and the Scherrer equation. The results are presented in Tables 1 and 2 and Supplementary Fig. 2, together with the errors associated with the values given, and analyses that assume size-only and strain-only broadening effects are shown for comparison. Rietveld analysis of control calcite crystals, which were 20–30 μm in size and were precipitated using comparable reaction conditions to those used for the calcite/polymer samples, gave a best fit with a domain size of 870 nm and strain of 0.004%, whereas a Williamson–Hall plot yielded a size and strain of 678 nm and 0.006%. These crystals can be considered virtually strain-free, and thus the Scherrer equation predicts comparable domain sizes of 798–825 nm (depending on the diffraction peak used for analysis). In the case of 40 μm Co^2+^ calcite crystals, which are widely described as single crystals in which Co^2+^ is substituted for Ca^2+^ in the lattice^28^, Rietveld analysis yielded a domain size of 300 nm and strain of 0.128%, whereas Williamson–Hall gave an (unphysical) −1,812 nm for the size and a strain of 0.21%. This negative-domain size arises because intrinsic errors in the data can cause the Williamson–Hall plot of crystals with large domain sizes to intercept the *y* axis at a small negative value. As the intercept is inversely proportional to the domain size, a large negative-domain size is returned. Application of the Scherrer equation, in contrast, yielded domain sizes of 92 nm as broadening arising from lattice strain is attributed to reduced domain sizes. These diffraction data are therefore fully consistent with the description of a single crystal exhibiting considerable lattice strain.

Looking in turn at calcite crystals precipitated in the presence of the polymers, the calculated domain sizes and strain determined using the Rietveld and Williamson–Hall methods, respectively, were 553 nm/0.024% and 1,014 nm/0.049% for calcite/PSS-MA, 612 nm/0.035% and 3,228 nm/0.072% for seeded calcite/PSS-MA, and 622 nm/0.016% and 647 nm/0.021% for calcite/PS-MA crystals (Table 1). As these domain sizes are comparable to pure calcite, this shows that the  line broadening observed for the calcite/polymer samples is almost entirely due to lattice strain. This is also seen in the underestimate of the domain sizes determined for these samples using the Scherrer equation, which were 321/228 nm (calcite/PSS-MA), 278/185 nm (seeded calcite/PSS-MA) and 368/435 nm (calcite/PS-MA) for the {104} and {006} reflections, respectively. That the sizes estimated for the {006} reflections are generally smaller than those for the {104} is consistent with the greater strain present in the ‹001› direction, as demonstrated in the Williamson–Hall plots (Supplementary Fig. 2). This can be attributed to preferential adsorption of the polymer additives on {001} planes. These data conclusively demonstrate that all of the mesocrystals examined show comparable domain sizes (≈500 nm), and that the primary source of line broadening is microstrain, where this can arise from organic occlusions within the crystals^31^,^32^,^33^. Notably, the seeded calcite/PSS-MA crystals, which are 10–20 μm in size and contain a 3-μm pure calcite core, gave comparable data to the calcite/PSS-MA crystals, suggesting that they have similar structures.

It has been proposed that the structures of mesocrystals can change with ageing due to fusion of the nanoparticulate subunits or the crystallization of residual ACC within the structure^24^,^26^,^27^. These possibilities were studied here by analysing annealed and aged calcite/polymer samples. Freshly prepared calcite/PSS-MA crystals were isochronously heated for 30 min at temperatures of 100, 200 and 300 °C, and synchrotron PXRD patterns were recorded after cooling. A sample was also analysed after ageing in air for 24h. No changes in the intensities of the diffraction peaks, the domain sizes (from 446 to 519 nm) or microstrains (from 0.041 to 0.046%) were observed after any of these protocols (Table 2 and Supplementary Fig. 3). These analyses were further confirmed using Raman microscopy, where no change in the peak shapes or broadening were recorded (Supplementary Fig. 4). Even though there was no ACC phase observed at all in the pattern initially, as ACC sometimes required temperatures >300 °C to crystallize^34^,^35^, additional *ex situ* annealing experiments were performed over the temperature range ambient to 400 °C. Only >400 °C were any changes in the diffractograms observed, where these were apparent in increases in domain size (from 708 to 1,493 nm) and strain (from 0.025 to 0.039%; Table 2 and Supplementary Fig. 3c). This can be attributed to decomposition of the polymers at 350 °C (Supplementary Fig. 5), and similar behaviour has been observed for biogenic and synthetic calcite crystals occluding organic molecules^4^,^31^. Therefore, these studies provide no evidence for ACC in the samples, or for epitaxial fusion of nanoparticle subunits.

**Figure 3:**
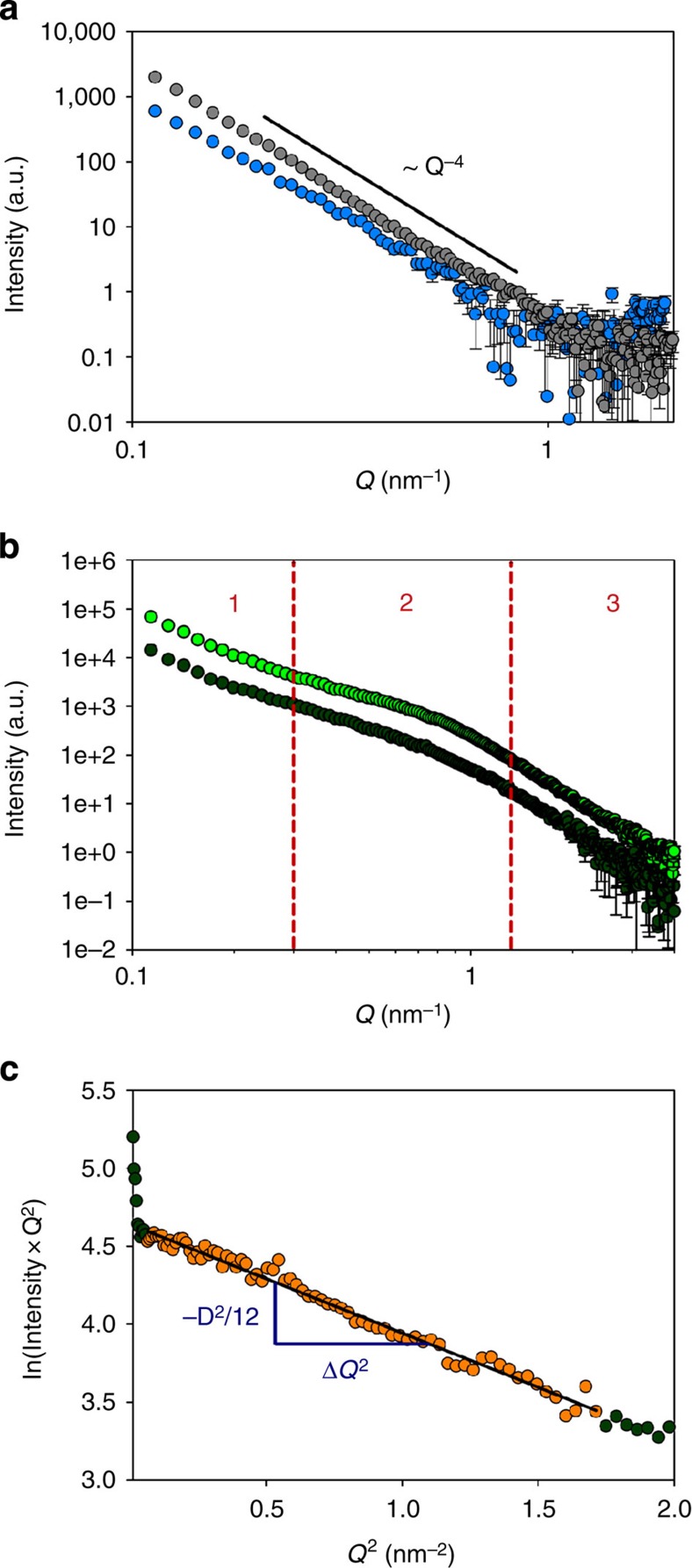
SAXS analysis. (**a**) Orientation-averaged plot of the scattering intensity versus the modulus of the scattering vector **Q** (log–log representation) recorded for powdered samples of control calcite (grey circles) and calcite crystals precipitated in the presence of Co^2+^ ions (blue circles, [Ca^2+^]:[Co^2+^]=50:1). The control sample shows an intensity decay of *I(Q)*∝*Q*^−4^ over the entire *Q*-range covered by the experiment, thus indicating the absence of structural complexity on the nanometre level. In the profile recorded for Co-doped calcite, there is a deviation from Porod-like behaviour in the low *Q* regime, which might be attributable to the rough-textured topography of the particle surfaces. (**b**) Orientation-averaged plots of the scattering intensity versus the modulus of the scattering vector **Q** (log–log representation) recorded for powdered samples of seeded calcite/PSS-MA crystals (light green circles) or calcite/PS-MA crystals (dark green circles). The profiles of both specimens provide indications for structural complexity at the length scale of nanometres. (**c**) Guinier plot ln(*I*(*Q*)·*Q*^2^) versus *Q*^2^ valid for dilute platelet-shaped scattering objects applied to the scattering curve of calcite/PS-MA crystals (dark green circles). The linear relationship in the *Q*-range 0.25 nm^−1^ <*Q* <1.3 nm^−1^ (orange circles) points to a platelet shape of the nanostructural heterogeneities. From the slope of the regression line (black) a mean thickness of the platelets of *D*=2.9 nm can be calculated. The error bars in **a** and **b** represent s.d. calculated according to Gaussian error propagation on the basis of the uncertainty of the intensity in each data point.

Additional information on the internal structures of the calcite samples was obtained using SAXS. The Co-calcite gave rise to a scattering profile consistent with little structural complexity, as shown by its similarity to that of a control calcite sample (Fig. 3a). The minor deviation from the pure calcite scattering curve is most likely because of increased surface roughness^36^. Looking in turn at the calcite/PS-MA and seeded calcite/PSS-MA samples, their radially averaged intensity profiles were similar to those of calcite/PSS particles previously studied using SAXS^36^ (Fig. 3b). The curves can be divided into three regimes, where scattering at low *Q* (regime 1) is dominated by a steep linear decay, owing to the large external facets of the powder grains, whereas smaller nanostructural features within the mineral particles give rise to a bent curve shape in regime 2. More detailed analysis of the scattering profile obtained from the calcite/PS-MA crystals based on a modified Guinier law shows that the nanostructural heterogeneities within these particles can be described as dilute platelets with average thicknesses of ≈2.9 nm (Fig. 3c). This would be consistent either with a classic mesocrystal in which individual mineral units are separated by organic layers^27^ or with a single crystal containing organic occlusions; SAXS cannot distinguish between the two. Finally, in the limit of high *Q* values (regime 3), the profiles show a linear decay that corresponds to smooth interfaces. A full evaluation of the SAXS data is provided in the Supplementary Note 2.

### TEM analysis of the internal structures of crystals

**Figure 4:**
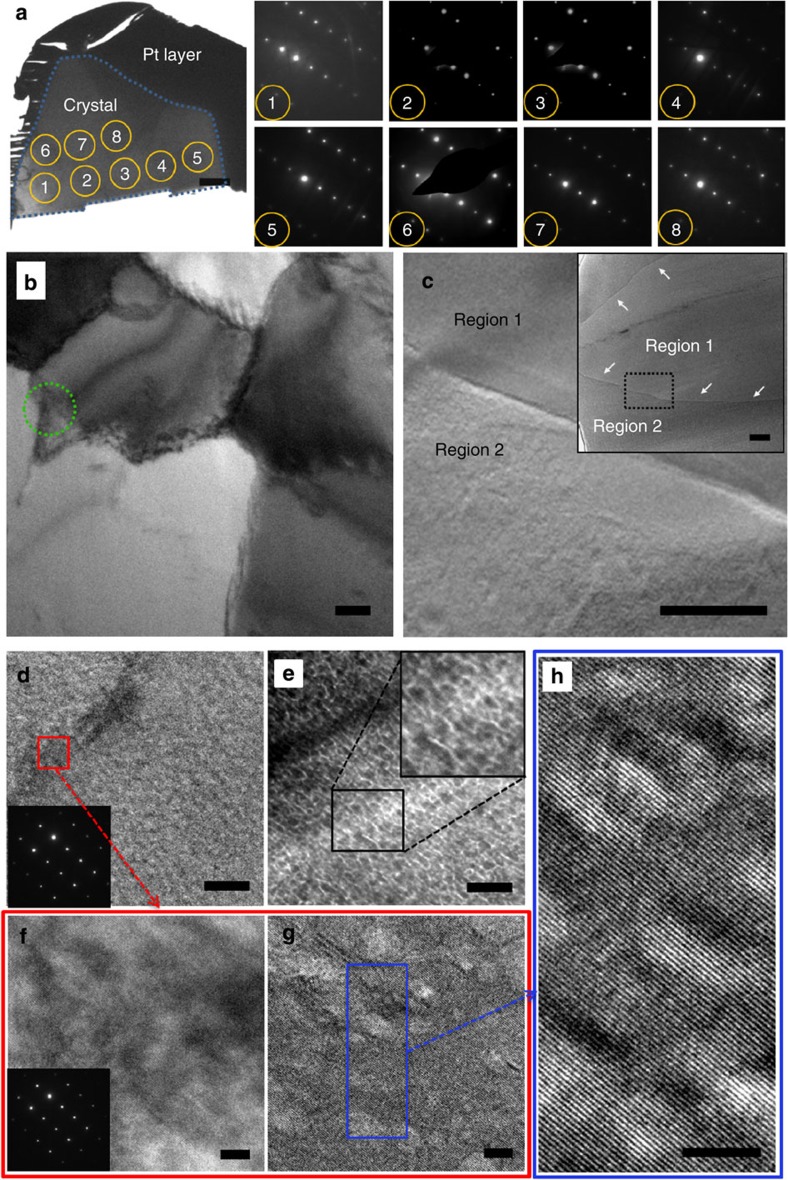
TEM images of thin sections of calcite/PSS-MA crystals. (**a**) Large FIB-section of a crystal grown at [Ca^2+^]=5 mM and [PSS-MA]=125 μg ml^−1^ (the blue outline indicates the boundary between the crystal and the Pt coating). The selected area electron diffraction patterns (from 1 to 8) were recorded across the whole crystal and show the single crystal structure. (**b**) A higher magnification image of the crystal shown in **a**, showing large mosaic blocks. (**c**) A crystal grown at [Ca^2+^]=1.25 mM and [PSS-MA]=125 μg ml^−1^ showing a central core and overgrowth layer, where the inset shows the same crystal at lower magnification. (**d**–**h**) High-magnification images of the area shown in the green circle in **b**. (**d**) An image taken at focus, which shows a continuous structure, and (**e**) the same area imaged significantly under focus, which shows an apparent nanoparticulate structure. (**f**) A higher magnification image of the area shown in the red box in **d** before beam damage, and (**g**) the same area after it has been beam damaged, resulting in porosity. (**h**) A HRTEM image of the area shown in the blue box in **g**, showing perfect lattice continuity, even after being beam damaged. Scale bars, 2 μm (**a**), 500 nm (**b**,**c**), 50 nm (**d**,**e**) and 5 nm (**f**–**h**).

The internal structure of calcite/PSS-MA crystals were investigated using TEM of thin sections cut using focussed ion beam (FIB), where samples were prepared immediately after removal of the crystals from solution. A large thin section cut through a crystal grown under conditions [Ca^2+^]=5 mM and [PSS-MA]=125 μg ml^−1^ is shown in Fig. 4a. This crystal exhibits a classic mosaic structure comprising 1–2 μm domains (Fig. 4b), and selected area diffraction patterns recorded over the entire area of the section (patterns 1–8) confirmed its single crystal structure. The size of these mosaic blocks is consistent with the >500nm domain sizes identified by PXRD. Calcite/PSS-MA crystals grown at lower initial supersaturations ([Ca^2+^]=1.25 mM and [PSS-MA]=125 μg ml^−1^) were also single crystals but displayed some differences in structure and comprised two distinct regions, namely a central core that appeared smooth and a rougher outer layer (Fig. 4c).

TEM has often been used to provide evidence for a nanoparticulate or porous sub-structure in mesocrystals. However, such structures can arise as artefacts, where these can be readily introduced into specimens during preparation of thin sections or during imaging itself. Roughness in the surfaces of the section can cause contrast variation in TEM images, which can be falsely interpreted as nanoparticle subunits (Fig. 4d,e)^37^. CaCO_3_ occluding organic additives is also particularly susceptible to beam damage^38^,^39^, and pores were generated in thin sections of synthetic calcite containing organic inclusions if they were not imaged with extreme care (Fig. 4f,g and Supplementary Fig. 6). Geological calcite is much less susceptible to beam damage (Supplementary Fig. 6). It is emphasized that no evidence for porosity and no discontinuity in lattice fringes were observed between the mosaic blocks (Fig. 4h).

### Analysis of surface areas and structures

High surface areas provide a further feature that is considered characteristic of mesocrystals, although an enormous range of values (varying from 48 to 540 m^2^ g^−1^) has been quoted in the literature for calcite mesocrystals^17^,^40^. It is also noted that an assessment of mesocrystal structure is very often made without measurement of the surface area, possibly owing to the relatively large quantities of sample required for accurate analysis. The surface areas and structures of calcite/PSS-MA and calcite/PS-MA crystals were determined, and the calcite/PSS-MA crystals were studied in detail (Supplementary Table 1). Comparison was also made with the surface areas of a number of reference samples including 20–30 μm synthetic calcite rhombohedra (0.1 m^2^ g^−1^), ground sea urchin skeletal plates (1–2 m^2^ g^−1^), 50–100 nm calcite nanoparticles (22 m^2^ g^−1^) and calcite precipitated in the presence of Co^2+^ ions (0.5 m^2^ g^−1^). The very low surface area of the latter is consistent with their description as single crystals^28^,^29^. The calcite/polymer particles, in contrast, showed large variations in specific areas (2–57 m^2^ g^−1^) depending on the initial solution supersaturation, the polymer concentration and the ageing conditions. Crystals with higher surface areas were obtained at higher initial supersaturations (SI_calcite_=3.43–4.17 gave surface areas of 1–4 m^2^ g^−1^, and SI_calcite_=5.76–6.47 gave surface areas of 25–60 m^2^ g^−1^), whereas PSS-MA concentrations of 150 and 300 μg ml^−1^ gave calcite crystals with surface areas of 40 and 56 m^2^ g^−1^, respectively. Crystals generated by overgrowth of a rhombohedral calcite seed at lower supersaturations also exhibited high surface areas of 28 m^2^ g^−1^.

**Figure 5:**
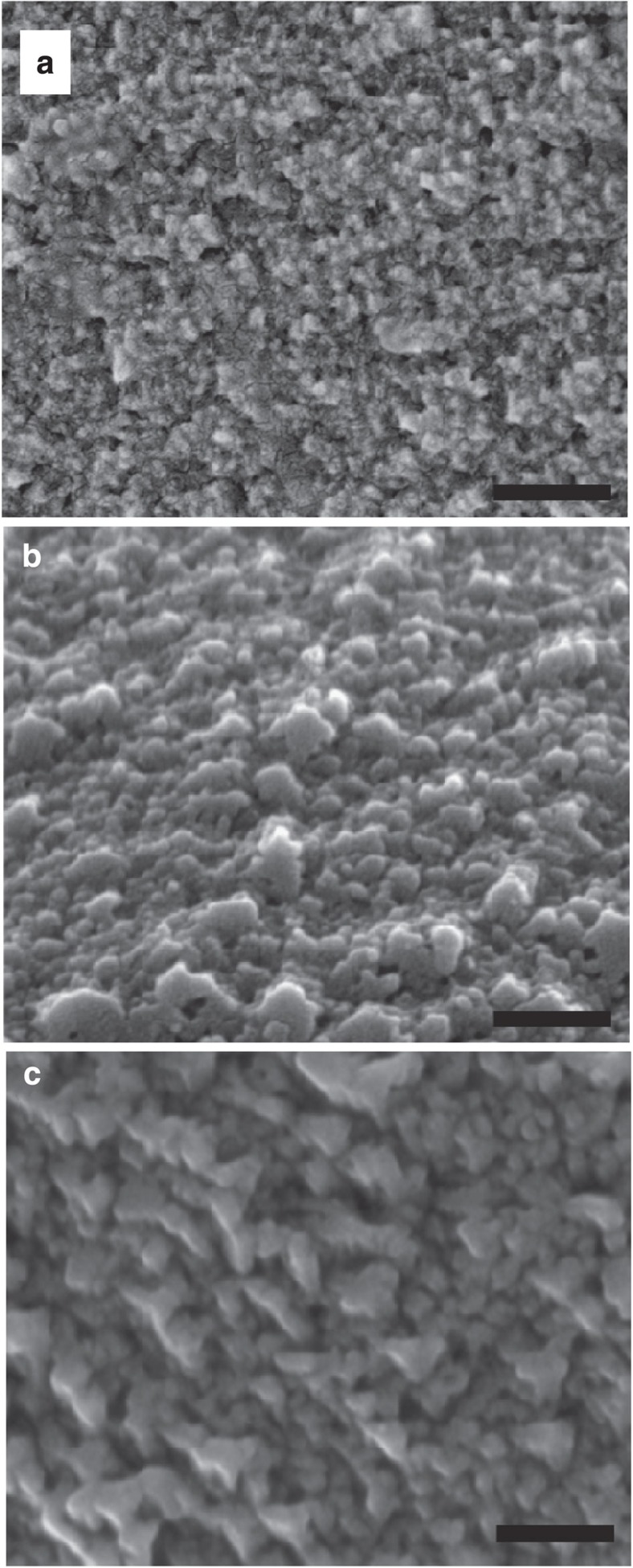
Effect of ageing on the surfaces of calcite/PSS-MA crystals. SEM images recorded immediately after (**a**) removing the crystals from the reaction solution after 1 day; (**b**) ageing the crystals for 2 days in air; and (**c**) incubation in the reaction solution without the carbonate source for 2 days. The precipitation was performed using the ammonium diffusion method (ADM) and the carbonate source was then removed. The images show that the surfaces of the fresh crystals are extremely rough, which gives rise to a high surface area. On ageing, these are replaced by much coarser features. Scale bars, 200 nm.

Ageing of dry calcite/PSS-MA samples in air had a marked effect on their surface areas, with reductions from 50–60 m^2^ g^−1^ to 5 m^2^ g^−1^ being observed after just 2 days under ambient humidity. In contrast, a much more gradual reduction in surface area was observed when samples were aged in the mother solution in the presence of ammonium carbonate, with a comparable reduction in surface area being observed over ≈2 weeks. That significant changes in the surface area occur on ageing the crystals suggests that the surface must recrystallize, changing the roughness. That this occurs more rapidly in humid air than in the original crystallization solution suggests that the residual polymer in the solution inhibits recrystallization. Indeed, calcite surfaces are well known to undergo reconstruction in water^35^, where this process will be modified in the presence of charged polymers^41^,^42^. The surfaces of calcite/PSS-MA crystals that had been freshly removed from the crystallization solution were therefore compared with crystals from the same batch after they had been aged in air for 2 days. The results are striking. The surfaces of the fresh crystals are covered with extremely small (<5 nm) particles (Fig. 5a), whereas the aged crystals exhibit much larger (30–40 nm) features (Fig. 5b). Crystals from the same batch were also examined after they had been incubated in the mother solution in the absence of ammonium carbonate for 2 days. Significant recrystallization was again apparent from the 80–90 nm geometric features viewed (Fig. 5c). This recrystallization process was also supported by thermogravimetric analysis of calcite/PSS-MA crystals. While crystals isolated after 1 day contained 4.1 wt% polymer, 10-day-old crystals occluded just 2.9 wt% polymer (Supplementary Fig. 5).

The nanoparticles on the calcite/polymer crystal surfaces are therefore the origin of the high surface areas sometimes measured. Using back-of-the envelope calculations to illustrate, a 10-μm calcite crystal with a 50-nm outer shell comprising 5 nm particles would exhibit a specific surface area of 63 m^2^ g^−1^. The fact that freshly prepared calcite/polymer crystals typically showed surface areas of 50–60 m^2^ g^−1^, which reduced to 5–20 m^2^ g^−1^ on ageing in air for longer than 2 days, is thus fully consistent with this model. It should also be noted that BET measurements are unreliable on small quantities of sample (Supplementary Table 2), which may also contribute to some of the variability observed in the literature.

### Electron backscatter diffraction analysis

**Figure 6:**
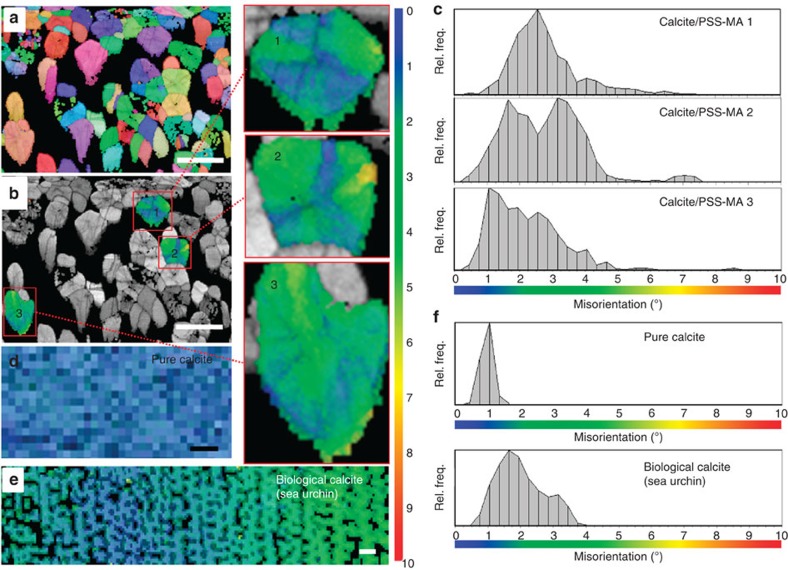
EBSD analysis. (**a**,**b**) EBSD maps of the calcite/PSS-MA crystals (measured with a raster step size of 280 nm); and (**c**) the histograms of the frequency distributions of misorientation for three selected calcite/PSS-MA individuals (1, 2, 3 in **b**). (**d**–**f**) These are compared with the EBSD maps and misorientation statistics for a calcite single crystal (**d**,**f**) and the spine of the sea urchin *P. lividus* (**e**,**f**). In **a**, the colour codes are for the absolute orientations of the crystals covering the entire orientation space. In **b**,**d**,**e**, the colour codes for misorientation with the colour scale defined on the right side of the maps and below the misorientation histograms (0° misorientation: dark blue, 10° misorientation: dark red). Superimposed on the colour maps is the EBSD band contrast, a grey scale component that gives the signal strength in each individual EBSD Kikuchi diffraction pattern. The band contrast highlights the grain boundaries and boundaries between mosaic blocks. For the calcite single crystal (**d**), the misorientation histogram corresponds to our experimental resolution of ±0.3° s.d. in crystal orientation. Scale bars, 20 μm (**a**,**b**,**e**) and 2 μm (**d**). Rel. freq., relative frequency.

Further information on the microstructures of the calcite/PSS-MA crystals was obtained using EBSD. The spine of the sea urchin *Paracentrotus lividus* (which has been described as a mesocrystal)^27^ and pure synthetic calcite were also analysed for comparison. Figure 6a,b shows the EBSD maps of these samples, together with histograms that show the misorientation for each EBSD-measured pixel (width 280 nm) relative to the mean orientation of each crystal. The EBSD maps of the calcite/PSS-MA crystals clearly show that they typically display primary mosaic structures that comprise sectors that radiate from the centre of a crystal (highlighted 1, 2 and 3 in Fig. 6b). This leads to a distribution of misorientation in the order of 5–7° (Fig. 6c). The growth sectors radiate from what appears to be a common substrate in the centre of the crystal, where the degree of mutual crystallographic misalignment is consistent with imperfect homoepitaxial overgrowth on a smaller calcite crystal. Interestingly, the orientational changes between the sectors are gradual rather than sharp and the distribution of orientations is diffuse in many places, which indicates orientation changes on a length scale of ≈500 nm. This is consistent with the size of the coherently scattering domain as determined by PXRD. Each mosaic sector also has an internal secondary mosaic distribution, which is larger than that of the pure calcite single crystal (the latter corresponds to our experimental resolution); Fig. 6d. The sea urchin spine (Fig. 6e), in contrast, exhibits a misorientation spread of about 4° (Fig. 6f) and shows a gradual change of orientation over the mapped area rather than sectoring. Occluded non-crystalline material in the form of pores and/or organic matrix in the sea urchin spine appears as dark areas in the map.

These data demonstrate that the calcite/PSS-MA crystals do not grow by a continuous process, and that growth was interrupted and re-started several times. Further, as the misalignments present in these crystals are absent in the pure calcite control, they must be ascribed to the effect of the polymer, which disturbs growth. These data also provide a valuable opportunity to compare the microstructures of the calcite/polymer crystals with that of a calcite biomineral. As structure informs the mechanical properties of biominerals, organisms exert strict control over crystallization processes and classic sector-zoning and small-angle boundaries between large mosaic blocks are rare. Instead, the sea urchin spine exhibits a uniform nanoparticulate structure with ubiquitous small-angle-misorientation fluctuations on the 100–200nm scale, where these are associated with intracrystalline organic matrix^43^,^44^,^45^. This structure then imparts considerable fracture resistance. Similar nanotextures have been observed in a range of calcite biominerals, where they are used to produce continuous orientation gradients and structures comprising interdigitated crystals^43^,^44^,^45^.

### Analysis of growth mechanisms

**Figure 7:**
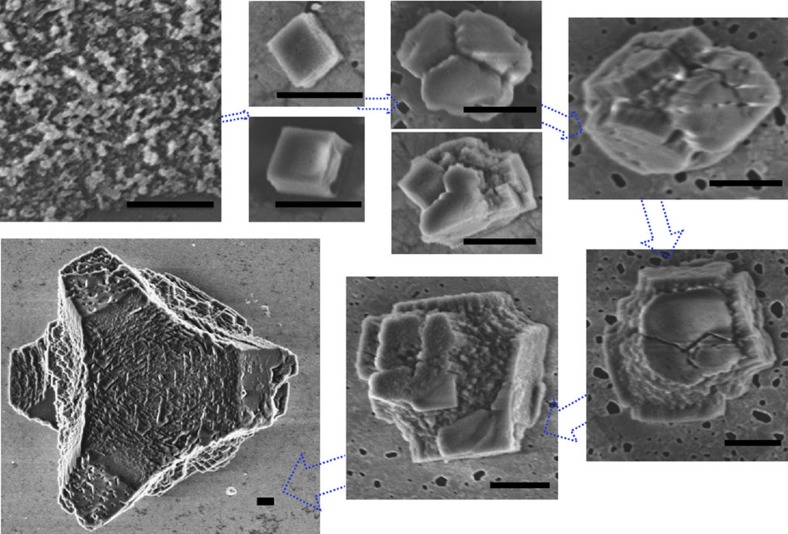
Morphological development of calcite/PSS-MA crystals. The crystals were precipitated in arrays of droplets formed on patterned self-assembled monolayers (SAMs) exhibiting arrays of 200 μm diameter circles in the presence of [Ca^2+^]=2.5 mM and [PSS-MA]=100 μg ml^−1^. The morphology of crystals developed from ACC phase to a fully grown crystal. Scale bar, 500 nm.

While ACC has often been observed as a precursor phase of calcite ‘mesocrystals’ precipitated in the presence of anionic polymers^17^,^46^,^47^, their full developmental pathway has remained unclear because of the problems associated with isolating rapidly growing crystals at precise points in their development. To address this challenge, we investigated the morphological evolution of the calcite/PSS-MA crystals by performing the crystallization reaction within picolitre droplets formed on patterned self-assembled monolayers^48^. Crystal growth terminates when the limited quantities of reagents within the droplets are depleted, revealing intermediate growth morphologies. Calcite/PSS-MA crystals were studied, and ACC was identified at early times (Fig. 7). Rather surprisingly, however, the first crystalline particles formed were 200–500 nm calcite rhombohedra. Only when the crystals had grown to sizes of 0.5–1 μm did modifications in morphology become apparent, and further growth then gave the characteristic pseudo-dodecahedral morphology of the calcite/PSS-MA crystals. These later growth stages are consistent with those reported for the growth of calcite/PSS-MA crystals at low polymer concentrations by Song *et al*.^47^, where a morphological transition from pseudo-dodecahedral morphologies to curved, concave surfaces was observed at particle sizes of ≈2–5 μm.

**Figure 8:**
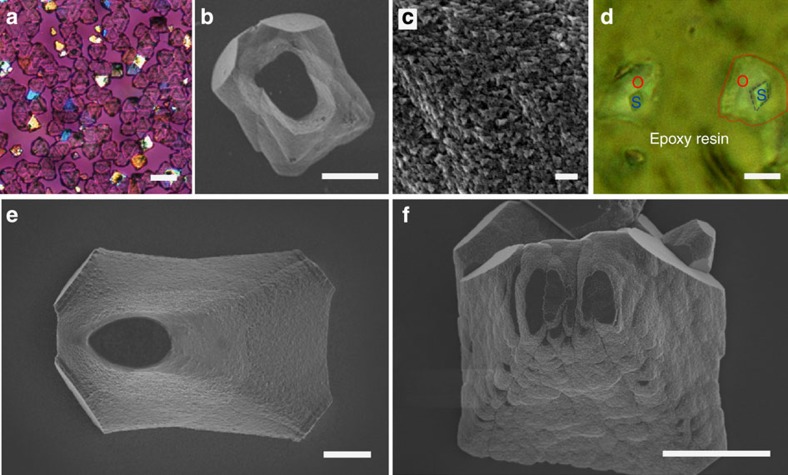
Crystal morphologies are not defined at early stages of growth. Images of calcite crystals produced by overgrowth of 3–5 μm calcite seeds at [Ca^2+^]=2.5 mM in the presence of [PSS-MA]=125 μg ml^−1^. (**a**) Optical micrograph; and (**b**) SEM image of a crystal produced by overgrowth; and (**c**) high-magnification SEM image of the surface of a crystal produced by overgrowth, showing a nanoparticulate structure. (**d**) Optical micrograph of mechanically polished crystals that had been embedded in resin, showing the overgrowth of calcite (labelled O) on rhombohedral cores (labelled S). Calcite crystals grown in the presence of PSS-MA from solutions that are undersaturated with respect to ACC. (**e**) [Ca^2+^]=0.5 mM, [CO_3_^2−^]=10 mM and [PSS-MA]=50 μg ml^−1^, SI_calcite_=3.49 and SI_acc_=−1.32; and (**f**) [Ca^2+^]=0.5 mM, [CO_3_^2−^]=200 mM and [PSS-MA]=50 μg ml^−1^, SI_calcite_=4.70 and SI_acc_=−0.19. Scale bars, 20 μm (**a**), 5 μm (**b**,**d**–**f**), and 200 nm (**c**).

Further confirmation that the characteristic mesocrystal morphologies are not determined at early growth stages was demonstrated through crystal overgrowth experiments in which calcite/PSS-MA was precipitated on 3–5 μm rhombohedral calcite seeds (Fig. 8). The final morphologies of the overgrown crystals and their rough surfaces—which appear to comprise nanoparticle units—were identical to those of crystals precipitated without seeds (Fig. 8a–c). These crystals also exhibited high surface areas (Supplementary Table 1), and mechanical polishing of overgrown crystals embedded in epoxy resin confirmed the overgrowth structure (Fig. 8d). The dimensions of the seed crystal itself also had some influence on its further morphological development, such that less change in morphology was observed when seeds >10 μm in size were used (Supplementary Fig. 7). This is readily explained as the larger the seed, the more material that is required to produce the same increase in thickness.

Finally, the influence of ACC as a precursor phase on the development of ‘classic’ mesocrystal morphologies was investigated by precipitating calcite/PSS-MA crystals from solutions that were very undersaturated with respect to ACC^49^ (Supplementary Table 3). While very few crystals were precipitated at initial supersaturations of sI_acc_≈−3.66 to −2.24 and SI_calcite_≈0.11 to 2.57, particles with characteristic pseudo-dodecahedral morphologies began to emerge at initial supersaturation levels of −1.76<SI_acc_<−0.45 and 3.05 <SI_calcite_<4.31, although they did not show the typical ‘scales’ on the surface which appear at higher supersaturations (Fig. 8e and Supplementary Fig. 8). The surface areas (1–4 m^2^ g^−1^) and polymer contents (1.3 wt%) of crystals grown under these conditions were also significantly lower than for those precipitated at higher supersaturations (Supplementary Table 1 and Supplementary Fig. 5). That these crystals—which were produced in the absence of ACC—exhibited the curved surfaces often associated with this phase is particularly interesting, where this demonstrates that care must be taken in deducing crystal growth mechanisms on the basis of final morphologies alone. With further increase in the supersaturation (but keeping solutions undersaturated with respect to ACC), the calcite/PSS-MA crystals developed more defined pseudo-dodecahedral morphologies and roughened surfaces (Fig. 8f and Supplementary Fig. 9). These experiments therefore provide strong evidence that development of these characteristic morphologies and rough surfaces does not depend on the assembly of crystalline nanoparticles.

## Discussion

In light of the data presented here, it is valuable to return to the early published work on calcite/polymer mesocrystals. On the basis of structural data, a suggestion was made that these crystals formed by the assembly of crystalline nanoparticles as mediated by adsorbed polymers. Although this mechanism has never been proven experimentally, and the definition of a mesocrystal has now been relaxed such that it is made purely on the basis of structure and not formation mechanism^27^, it rather caught the imagination. As a result, the current literature is still dominated by a belief that large single crystals can form by the assembly of crystalline nanoparticles, where the external morphology reflects the shape of the basic building block. The extensive and rigorous analysis of the structure and formation of calcite/polymer crystals described in this article contradicts this picture.

Let us first consider the structural data. While it is tempting to view a single crystal as one in which every atom lies in its perfect position, the reality is that almost all crystals are imperfect. Imperfect crystals have been discussed since the early 1900s, when it was recognized that measured diffraction intensities depend on crystal perfection^50^. While the diffraction intensities in a perfect crystal are proportional to the structure factor F and have angular spreads in the order of seconds of arc, most crystals give intensities orders of magnitude higher with angular spreads of minutes of arc. Attempts to rationalize these erroneous diffraction intensities were therefore made using a range of models to describe imperfect crystals. One of the earliest was that of a ‘mosaic crystal’^51^, where this envisaged a crystal as a mosaic of perfect crystalline blocks that are slightly misaligned with respect to each other. These misorientations can destroy the coherence between radiation reflected from different depths of the crystal, resulting in an enhancement of the reflected intensity. Although originating as a mathematically tractable model, the concept of a mosaic crystal appears to apply quite well to many natural crystals that comprise micron-sized blocks^52^.

A significant advance in the understanding of imperfect crystals was then made with the recognition that crystals contain dislocations—atomic-scale defects—and that simple crystal boundaries can be described in terms of arrays of dislocations^53^. A complete description of a crystal and its diffraction behaviour thus depends on knowledge of the type and positioning of the imperfections present. Common defects/sources of strain in crystals include dislocations, stacking faults, twinning, grain boundaries, chemical heterogeneities and inclusions, where these can manifest themselves in characteristic changes in peak positions, broadening and shape^54^. Full-pattern analysis, considering the position, width and shape of the peaks can provide some insight into the nature of imperfections in crystals, where dislocations, for example, can contribute to line broadening owing to their mean separation (which is inversely proportional to their density) and microstrains arising from internal stress fields^55^. That line broadening due to structural errors can also vary with *hkl* according to the type of fault present (for example, stacking faults or twins) also provides a further source of information.

A further model of crystal imperfection is that of paracrystallinity, where this has been used to describe structures that are intermediate between crystalline and amorphous^56^. Indeed, this model is often used to interpret the structure of polymers^57^, and bone has been described as a paracrystalline material^58^. Analysis of our diffraction data from the calcite/PSS-MA crystals provides no evidence for paracrystallinity, where this is entirely expected given that calcite is well recognized to form exceptionally large, perfect crystals^53^. The detailed analyses of the high-resolution synchrotron XRD data instead conclusively show that the observed line broadening arises from microstrains within the crystal lattice rather than small domain sizes. We attribute these to the incorporation of polymer within the crystal lattice, as is consistent with the SAXS data. This causes a distortion of the lattice in their vicinity, resulting in a distribution of tensile and compressive forces. Indeed, this effect has also been observed for calcite biominerals^31^,^32^,^33^, and for calcite single crystals occluding amino acids^5^ and 20 nm block copolymer micelles^4^. Previous analysis of calcite/PSS crystals also yielded comparable data, which like the calcite/PSS-MA and calcite/PS-MA crystals, demonstrated preferential adsorption of the polymer on {001} planes^36^. The absence of shifts in the peak positions demonstrates the absence of uniform macrostrains.

Considering then the mechanism of formation of the calcite/polymer crystals, our data again provides no evidence for the assembly of crystalline precursor particles. In showing that calcite rhombohedra form before the characteristic ‘mesocrystal’ morphologies, and that these morphologies can also develop on overgrowth of calcite seed crystals, we demonstrate that the shape is not defined at nucleation. Instead, a morphological transition does not occur until the rhombohedra reach sizes of 0.5–1 μm. This can be explained by the change in the solution conditions with time. The morphologies of calcite crystals precipitated in the presence of soluble additives are determined by both kinetic and thermodynamic factors, where changes in the shape and separations of the atomic terraces dictate the macroscopic changes in the crystal morphology^59^,^60^. Further, it has also been observed that polymeric additives can modify the crystallization pathway of calcite from the typical step flow at dislocations that dominates at supersaturations of SI <0.8 (ref. 61) to two-dimensional nucleation and growth on the terraces^62^. Two-dimensional nucleation is expected at the supersaturation levels used here, and is consistent with the crystal morphologies and mosaic blocks observed. As the polymer/Ca^2+^ ratio in the growth solution will increase with time because of the much higher rate of depletion of the Ca^2+^ ions than the polymer—while a near-constant supersaturation level is maintained using the ammonia diffusion method^35^—the effect of the polymer will become more significant. The nanoparticulate surfaces observed towards the termination of crystal growth are likely to arise owing to the rapid decrease in supersaturation that occurs at this stage of the ammonia diffusion method. With high polymer/[Ca^2+^] ratios and a low supersaturation, surface poisoning by the polymer additive will become more significant^35^.

ACC has also been frequently observed at early stages of formation of calcite/polymer mesocrystals, suggesting that ACC may be an essential precursor phase. Indeed, growth of crystals by transformation of an amorphous precursor phase has marked parallels with the formation of biogenic mesocrystals such as sea urchin spines^27^. Running counter to this argument, our data show that calcite crystals that morphologically resemble `classic' mesocrystals can be precipitated in the presence of polymer, in solutions that are well below the saturation level of ACC. Further, these crystals can exhibit asymmetric morphologies and curved surfaces, demonstrating that such morphologies can actually occur by classical growth mechanisms. This is fully consistent with alternative studies that have demonstrated that CaCO_3_ particles with morphologies identical to reported vaterite mesocrystals can be formed below the ACC supersaturation level and in the absence of additives through control of the supersaturation alone^63^. These studies also demonstrated that supersaturation values orders of magnitude higher would be required to generate sufficient numbers of nanoparticles to support a growth mechanism based on the aggregation of crystalline nanoparticles^64^. However, given that the majority of calcite/polymer crystals studied here were precipitated at an initial supersaturation level where ACC formation is expected, it is likely that a range of crystal growth mechanisms operate during their transition from nuclei to crystals of over 10 μm in size.

In conclusion, the detailed analysis of calcite crystals presented here provides a clear demonstration that great care needs to be taken when classifying particles as mesocrystals. Indeed, while observations of nanoparticulate surface structures, high surface areas, line broadening of PXRD spectra and characteristic morphologies are all routinely used to assign mesocrystal structure, none of these provide stand alone evidence for the nanoparticulate sub-structure that is now considered to define a mesocrystal^27^. The misinterpretation of PXRD is particularly widespread, and we re-emphasize that the Scherrer equation cannot be used to estimate particle sizes when lattice strain is also present (as is often the case when crystals are co-precipitated with additives). We also reiterate that our work provides no evidence for the formation of these calcite/polymer crystals by the assembly of crystalline precursor particles, and that morphologies that are considered signatures for mesocrystals can also be generated in the absence of an ACC precursor phase. In moving on, it is therefore essential that researchers are rigorous in their analyses of crystals, and that the many articles describing not only CaCO_3_ but also other types of mesocrystals are re-examined, especially where assembly-based formation mechanisms have been proposed.

## Methods

### Materials

CaCl_2_·2H_2_O, (NH_4_)_2_CO_3_ and CoCl_2_·6H_2_O were purchased from Sigma-Aldrich and were used without further purification. PSS-MA and PS-MA were purchased from Sigma-Aldrich and were used after purification by dialysis against copious amounts of water for 2 days to remove impurities, followed by freeze-drying.

### CaCO_3_ precipitation

Calcium carbonate was precipitated in the presence of the soluble anionic block copolymers PSS, PSS-MA and PS-MA. Two precipitation methods were used, such that CaCO_3_ was precipitated using either the ammonium carbonate diffusion method or by a double decomposition method (precipitation from a metastable solution) where equal volumes of [(NH_4_)_2_CO_3_]=1–100 mM solution and [CaCl_2_·2H_2_O]=0.2–5 mM solution were added in a Petri dish and then incubated at room temperature for 24 h. Of these, the ammonium carbonate method was principally used, and was used unless stated otherwise.

CaCO_3_ was precipitated in the presence of the polymers in a polystyrene dish containing 10 ml of solution. Glass substrates were cleaned by soaking in Piranha solution (70:30 H_2_SO_4_:H_2_O_2_) before use, and were placed at the base of the dish. Stock solutions of polymers were added to a solution of [CaCl_2_·2H_2_O]=0.5–5 mM to form final solutions with polymer concentrations of 50–300 μg ml^−1^. Precipitation of CaCO_3_ was then initiated by placing the dish in a sealed desiccator containing a glass Petri dish of (NH_4_)_2_CO_3_ (5 g), which was covered with a piece of parafilm pierced four times with a needle. Crystallization was typically allowed to proceed for 1–2 days (unless stated otherwise). Following this period, the glass slides supporting the CaCO_3_ crystals were removed from the solutions, washed with Millipore water and dried in air.

For comparison with the crystals generated in the presence of copolymers, CaCO_3_ was also co-precipitated with cobalt (II) ions using the Kitano method^65^. In brief, CO_2_ gas was bubbled through a saturated suspension of CaCO_3_ for 3–4 h before filtering (200 nm, Millipore filters) to remove any undissolved CaCO_3_ particles. CO_2_ gas was then bubbled through the solution for a further 10–20 min. CoCl_2_·6H_2_O was then added to the prepared Kitano solution to give a final Co(II) concentration of 0.01–2 mM, and precipitation was induced by opening the prepared solution to air to allow escape of CO_2_ gas^28^.

### Overgrowth on seed calcite crystals

Overgrowth experiments were performed on seed calcite crystals either 3–5 μm or 10–30 μm in size. Rhombohedral calcite seed crystals were precipitated on mica or glass substrates using the ammonia diffusion method as described above. After brief washing and drying, the substrates supporting the crystals were transferred to a solution of 2.5 mM CaCl_2_·2H_2_O and the desired soluble polymer, and precipitation was carried out via ammonium diffusion for 1 day, before isolating from solution. The overgrown crystals were then scraped from the substrate using a razor blade, dispersed in epoxy resin and subsequently cured on a silicon wafer. The specimen was then mechanically polished and was examined using optical microscopy and SEM.

### Precipitation of calcite crystals in droplet microarrays

Experiments were performed using established methods^48^. In brief, 50 nm Au films were deposited onto freshly cleaned glass substrates after pre-deposition of 2–5 nm of Cr to promote adhesion between the glass and the Au. The Au surfaces were then functionalized with hydrophobic self-assembled monolayers of 1H,1H,2H,2H perfluorodecane thiol (Sigma-Aldrich, 99%) by immersing the substrate in dichloromethane solutions of 0.1 mM perfluorodecane thiol at 4 °C for 24 h. The substrates were rinsed in ethanol before subsequent exposure to a deep UV light source (*λ*≤255 nm, h*ν*>4.8 eV) through a circular quartz photomask at a constant distance of 2 cm for 1 h. The mask was patterned with an array of 100–200 μm diameter circles with centre-to-centre spacings of 100 μm. Photocleaved alkylthiols were then displaced by back-filling in dichloromethane solutions of 0.1 mM mercaptohexadecanoic acid (Sigma-Aldrich, 99%) for 1 h at 4 °C, before rinsing thoroughly in deionized water (18.2 MΩ cm, Milli-Q) and drying under a nitrogen gas stream.

CaCO_3_ precipitation was then carried out within a sealed chamber in which the humidity was controlled at 100%. A solution of [CaCl_2_·2H_2_O]=1.25–5 mM and [PSS-MA]=125–500 μg ml^−1^ was poured over a freshly prepared substrate, resulting in the formation of picolitre-volume droplets on the hydrophilic domains. The substrate was then placed in the equilibrated humidity chamber along with 0.1 g solid ammonium carbonate, and precipitation was allowed to proceed for up to 30 min, before removing the substrate, washing with ethanol and air-drying. Different growth stages were assessed by analysing particles present within different droplets at the same time, and over a range of times.

### Determination of supersaturations

The supersaturations of the crystallization solutions were calculated using visual MinteQ software, where the supersaturation index is defined as:


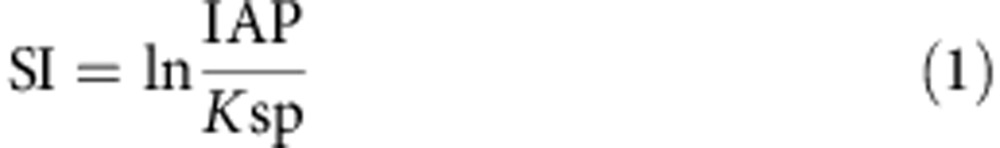


where IAP=ion activity product, *K*_sp_=solubility constant, *K*_sp_ (calcite)=10^−8.48^ and *K*_sp_ (ACC)=10^−6.39^.

The actual Ca^2+^ concentrations in the reaction solutions containing PSS-MA polymers were measured with a Ca^2+^ ion-selective electrode (Metrohm). All solutions were freshly prepared before each experiment using fresh Milli-Q water (resistivity 18.2 MΩ cm at 20 °C) and the electrode was calibrated with four standard CaCl_2_·2H_2_O solutions with concentrations in the range 0.5–10 mM. The desired concentrations of pure CaCl_2_·2H_2_O solutions were first measured, then CaCl_2_·2H_2_O solutions containing PSS-MA polymer were measured after stirring for 30 min. The (Davies-extended) Debye–Huckel equation was used to convert the measured Ca^2+^ activities into concentrations for determination of the activity coefficients.

### Characterization of CaCO_3_ particles

The CaCO_3_ particles were analysed using SEM, high-resolution TEM (HRTEM), optical microscopy, Raman microscopy, infrared spectroscopy, thermogravimetric analysis, surface area analysis (BET), atomic absorption spectroscopy and EBSD. Selected samples were also analysed using synchrotron PXRD and SAXS. For SEM, particles were typically transferred onto glass coverslips that were subsequently mounted on aluminium stubs using carbon sticky pads. Samples were then coated with 5 nm Pt/Pd using an Agar High Resolution Sputter Coater and were examined using a LEO 1530 Gemini FEG-SEM operating at 3 kV using an in-lens detector mode. For TEM analysis, an FEI Tecnai TF20 FEG-TEM fitted with Oxford Instruments INCA 350 EDX system/80 mm X-Max SDD detector and Gatan Orius SC600A CCD camera operating at 200 kV was used. Raman microscopy and infrared spectroscopy were used to further confirm the polymorph and internal structure change of crystal particles with Raman being carried out using a Renishaw 2000 Raman microscope operating with a 785-nm diode laser, and infrared being performed with a PerkinElmer ATR-IR. The Co content of samples was analysed using a PerkinElmer Atomic Absorption Spectrometer, AAnalyst 400 with an air-acetylene flame after dissolving samples in dilute HNO_3_.

### Surface area analysis (BET)

Surface area analysis was conducted using N_2_ absorption with a Micromeritics-TriStar 3000 after degassing for 2 h and/or heating up to 80 or 300 °C. The surface area was calculated from the linear part of the BET plot while the pore-size distribution was determined using the Barrer–Jovner–Halenda model. For multi point (5 point) BET measurements, the data were treated according to the absorption isotherm given in equation (1).


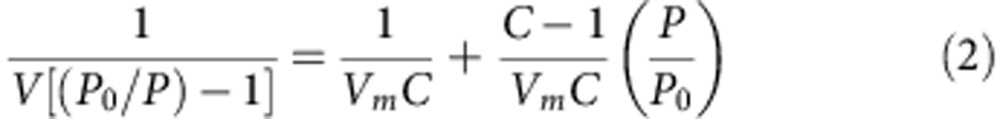


where *P*=partial vapour pressure of adsorbate gas in equilibrium with the surface at 77.4 K (boiling point of liquid nitrogen), in pascals, *P*_o_=saturated pressure of adsorbate gas, in pascals, *V*=volume of gas adsorbed at standard temperature and pressure (273.15 K and atmospheric pressure (1.013 × 10^5^ Pa)), in millilitres, *V*_*m*_=volume of gas adsorbed at standard temperature and pressure to produce an apparent monolayer on the sample surface, in millilitres, *C*=dimensionless constant that is related to the enthalpy of adsorption of the adsorbate gas on the powder sample.

A value of *V* was measured at each of 5 values of P/Po. Then, the BET value:


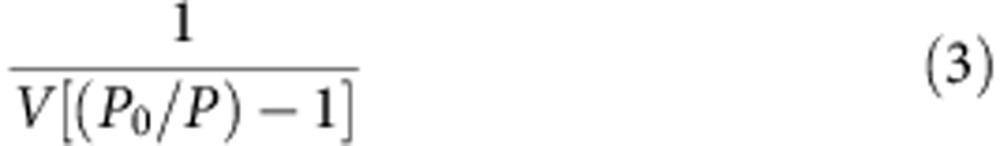


was plotted against *P*/*P*_o_ according to equation (2). This plot should yield a straight line in the pressure range ≈0.05–0.3. The data are considered acceptable if the correlation coefficient of the line is not <0.9975; that is, *r*^2^ is not <0.995 from the resulting linear plot, where the slope and intercepts are obtained as:


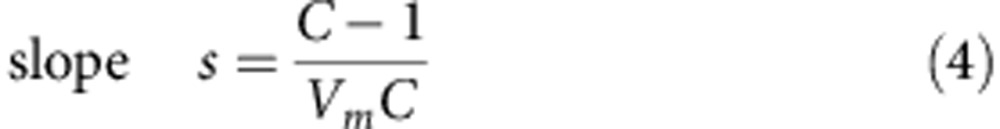



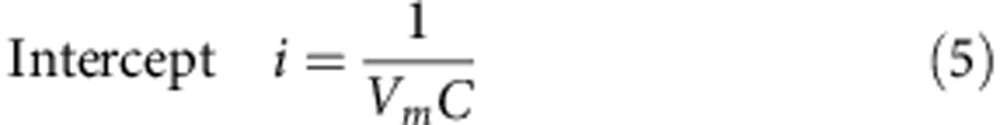


From this value, *V*_*m*_ is calculated as:


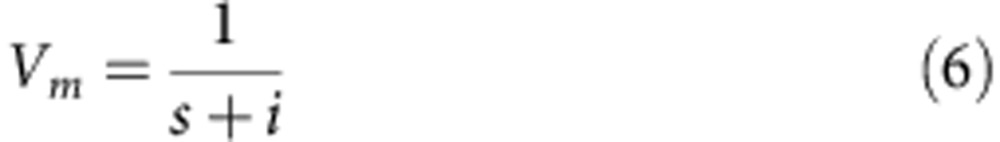


From the value of *V*_*m*_ determined, the total surface area *S*_*t*_ is calculated by the equation:


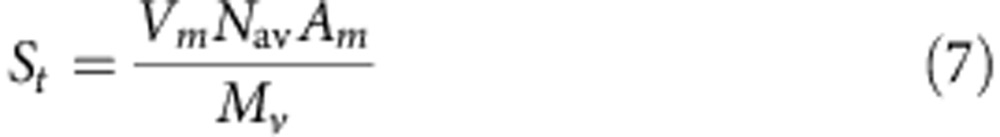


*N*_av_=6.022 × 10^23^, *A*_*m*_=0.162 nm^2^, *M*_*v*_=22 414 ml

Finally, the specific surface area *S* is obtained as:


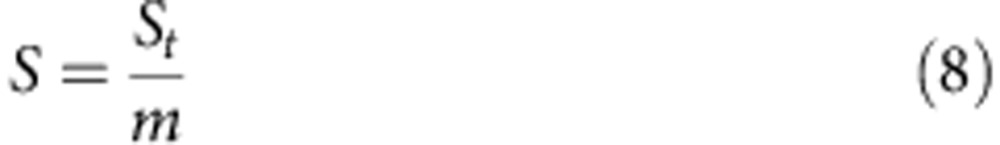


where *m*=the mass of sample used.

### FIB and HRTEM analysis

Images of the lattice structure of the calcite particles were obtained using HRTEM imaging of thin sections prepared by FIB Milling . FIB to electron transparency was performed using an FEI Dual Beam system equipped with a 30 kV Ga beam and a field emission electron gun operated at 5 kV. The samples were then analysed with a FEI Tecnai TF20 FEG-TEM fitted with an Oxford Instruments INCA 350 EDX system/80 mm X-Max SDD detector and a Gatan Orius SC600A CCD camera operating at 200 kV. In order to minimize electron beam related damage during the recording of images and diffraction patterns recording, a 10-μm condenser lens and the smallest spot size were used.

### Synchrotron PXRD studies

The high-resolution PXRD measurements were carried out at the dedicated high-resolution powder diffraction beamline (I11) at the Diamond Synchrotron Radiation Facility (Diamond Light Source Ltd, Didcot, UK). The beamline is equipped with a crystal monochromator (a liquid nitrogen-cooled double-crystal silicon monochromator) and a crystal analyser at the incident and diffracted beams, respectively. The optics of the diffracted beam consists of nine (111) Si crystal analysers and the use of the advanced analysing optics yielded diffraction spectra of superior quality that exhibited intense and extremely narrow diffraction peaks with an instrumental contribution to the peak widths not exceeding 0.004° (ref. 66). Instrument calibration and wavelength refinement were performed with silicon standard samples from the National Bureau of Standards and Technology (NIST; Gaithersburg, MD, USA). Powders for analysis were loaded into 0.7 mm borosilicate glass capillaries, and to avoid intensity spikes from individual crystallites, the samples were rotated during measurements at a rate of 60 r.p.s. using high-resolution multi-analyser crystal diffraction scans, with scan times of 1,800 s. Spectra were recorded both at room temperature and after *in situ* heating of specimens to temperatures of 100, 200 and 300 °C for 30 min using an internal heater.

### XRD analysis

The structural parameters were refined by Rietveld analysis both using GSAS and using PANalytical X’Pert HighScore Plus software. There was no evidence of amorphous material, as indicated by a flat baseline in the entire range 2*θ*=3–150°. Strain and size analysis was performed using both Rietveld analysis and line profile analysis. In order to quantify the broadening, Williamson–Hall plots were also prepared. Using this technique, B cos*θ* was plotted against sin*θ* for the {104}, {001} and {100} families of planes. The average microstrains and size effects (or coherence lengths) of each sample were determined from the gradient and intercept of the plot, respectively.

### Rietveld size/strain analysis

At least 20 reflection peaks were refined using a Pseudo-Voigt function and the size and strain analysis was plotted using the Caglioti equation^67^:


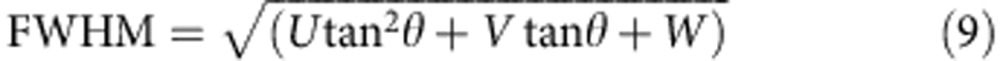


The *U* parameter contains the information about the strain broadening, whereas the *W* parameter corrects for a possible size broadening. Microstrain and size are then obtained using the following algorithm:

Microstrain is described by:


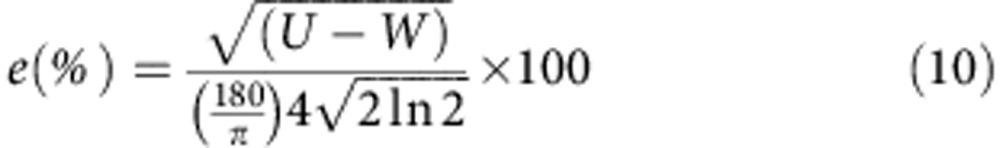


Crystallite size is described by:


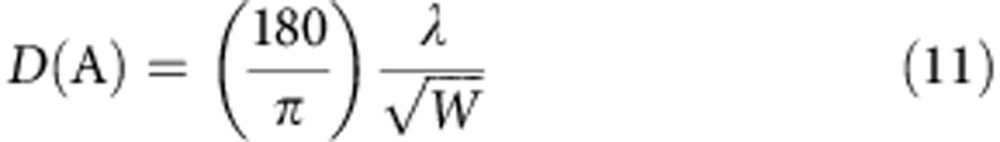


### Williamson–Hall analysis

The XRD data was also analysed by combining a Williamson–Hall plot with line profile analysis. This method uses the integral breadth as a measure of the peak width and the universal shape factor *Φ* to describe the peak shape variation. These numbers can be determined for single peaks as well as for a whole range of peaks. The algorithm then empirically deconvolutes the profile into a Gaussian and a Lorentzian integral breadth: This is defined as net peak area/peak height, and is equal to a rectangle with the same height and the same area as the net peak area; FWHM (2*θ*) is defined as the full-width at half-maximum intensity of the peaks. The universal shape parameter is defined as FWHM/integral breadth. The sample contribution to the total broadening is calculated using an empirical convolution of the Gaussian and the Lorentzian parts, where the Lorentzian broadening (*B*_L_) is given as:


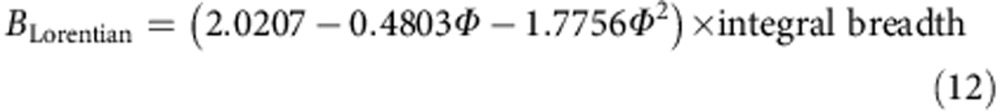


The Gaussian broadening (*B*_L_):


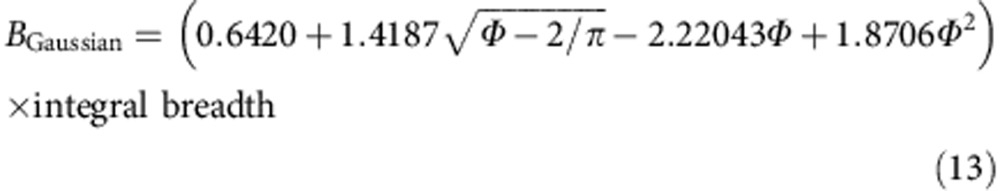


In order to construct the W–H plot, both Gaussian and Lorentzian broadenings are combined using the empirical convolution, where B cos*θ* is plotted against sin*θ*.


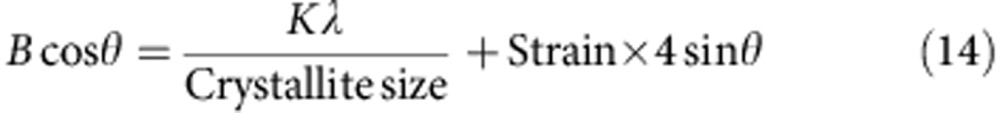


The strain(%) is then obtained from the slope (=4 × strain)

The crystallite size (Å) is obtained from:


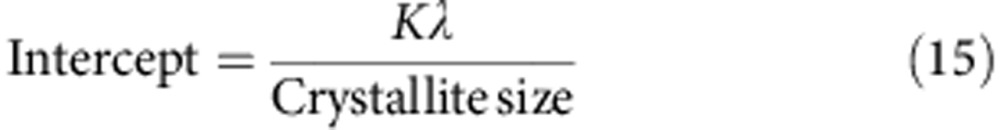


A shape factor of *K*=1 was used.

### Scherrer size analysis

The crystal size was also estimated using the Scherrer equation:


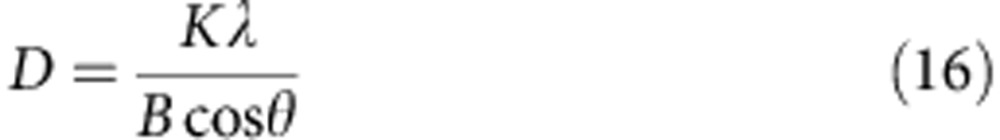


*B*=FWHM or integral breadth and a shape factor (or Scherrer constant) of *K*=1 was applied.

The integral breadth and FWHM as a measure of the peak width of selected peaks were used in the Scherrer equation. The individual peaks were fitted by line profile fitting as described above.

### Small-angle X-ray scattering

SAXS profiles of powdered samples (control calcite, Co^2+^-doped calcite, seeded calcite/PSS-MA crystals and calcite/PS-MA crystals) were recorded using a Nanostar Instrument (Bruker AXS, Karlsruhe, Germany) equipped with a single-photon counting area detector (HiStar, Bruker AXS). The X-ray generator provided radiation with a wavelength of *λ*=1.54 Å (Cu–*K*_α_) and was operated at 40 kV and 35 mA, while a parabolically bent graded multilayer (Göbel mirror) was used to ensure a parallel and monochromatic X-ray beam. All specimens were measured in borosilicate glass capillaries at a sample-detector distance of 105 cm. Scattering data for the polymer-containing particles were additionally recorded at a smaller sample-detector distance of 26 cm in order to cover a larger range of accessible values for the scattering vector **Q**. The integrated profiles of the scattering intensity versus the modulus of the scattering vector *Q* (*Q*=4π·sin(2*θ*)/λ), where 2*θ* is the scattering angle and *λ* represents the wavelength of the incident beam) were corrected for instrument-related background and sample transmission as well as scattering attributable to the sample container. The Laue background (*I*_Laue_) obtained from a Porod fit of the profiles (
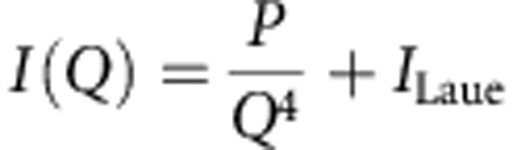
, *P* being the Porod constant) was subtracted from the data. For the crystal/polymer samples a *T*-parameter analysis was performed according to the method described by Fratzl *et al*.^68^ However, due to the strong scattering contribution of the large external surfaces of the powder grains in the low *Q* limit (*Q*<0.3 nm^−1^ for calcite/PSS-MA crystals and *Q*<0.25 nm^−1^ for calcite/PS-MA crystals), data points in this *Q*-range were not included in the trapezoidal integration of the Kratky curve (*I*(*Q*) *Q*^2^ versus *Q*), but were approximated by a rectangle curve.

### Electron backscatter diffraction

EBSD requires that samples have highly smooth surfaces, where this was achieved here by sectioning and polishing the crystals with an ultramicrotome (Leica). The surface of the sample was then coated with 4–6 nm of carbon. EBSD maps were obtained at 15 and 20 kV on a FEG-SEM (JEOL JSM 6400) equipped with an Oxford Instruments NordlysNano EBSD detector and interpreted by CHANNEL 5 EBSD software.

## Author contributions

Y.-Y.K. led the experimental work, preparing samples and carrying out TEM, BET and XRD analyses; A.S.S. performed the SAXS experiments, analysed the SAXS data and participated in sample preparation for EBSD; J.I. participated in the BET study of surface areas, whereas A.N.K. carried out the FIB preparation of samples and assisted with the BET analysis; C.C.T. provided access to Beamline I11 at Diamond and assisted with the XRD experiments; G.H. assisted with analysis and discussion of the XRD data; E.G. and W.W.S. performed the EBSD studies and analyses; N.B.J.H. performed early studies that inspired this work; F.C.M. originated and supervised the project. All authors contributed to the preparation of the manuscript.

## Additional information

**How to cite this article:** Kim, Y.-Y. *et al*. A critical analysis of calcium carbonate mesocrystals. *Nat. Commun.* 5:4341 doi: 10.1038/ncomms5341 (2014).

## Supplementary Material

Supplementary Information Supplementary Figures 1-9, Supplementary Tables 1-4, Supplementary Notes 1-2 and Supplementary References

## Figures and Tables

**Figure 1:**
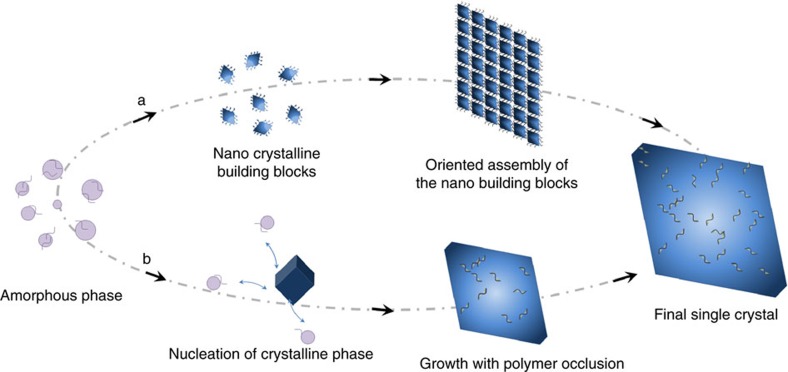
Schematic of calcite crystal growth mechanisms. (a) The original mechanism proposed to lead to calcite mesocrystal formation, based on the oriented assembly of crystalline nanoparticles; and (b) the mechanism demonstrated in this paper, where a calcite rhombohedron initially forms, and subsequent growth in the presence of polymer results in a modified morphology and a rough, particulate surface.

**Table 1:** Strain parameters and coherence lengths of calcite crystals.

	**Calcite control, 10 mM**	**Co-calcite**	**PSS-MA**	**PSS-MA seeded**	**PS-MA**
Rietveld (pseudo-Voigt)					
Size only (nm)	817 (32)	86 (7)	380 (34)	319 (24)	485 (15)
Strain only (%)	0.001 (2)	0.130 (3)	0.030 (4)	0.039 (3)	0.022 (4)
Size (nm) and strain (%)	870 (22)/0.004 (1)	300 (14)/0.128 (6)	553 (23)/0.024 (3)	612 (19)/0.035 (2)	622 (12)/0.016 (1)
Williamson–Hall plot					
Size only (nm)	446.4 (27)	54 (5)	141 (7)	109 (5)	243 (8)
Strain only (%)	0.017 (1)	0.200 (2)	0.057 (2)	0.074 (2)	0.0325 (7)
Size (nm) and strain (%)	678 (19.3)/0.006 (5)	−1,099 (2532)/0.210 (1)	1,014 (465)/0.049 (8)	3,228 (395)/0.072 (8)	647 (112)/0.021 (2)
Scherrer equation (104)					
Size only (nm)	825 (89)	92 (9)	321 (32)	278 (48)	368 (43)
Scherrer equation (006)					
Size only (nm)	798 (56)	NA	228 (25)	185 (21)	435 (56)

NA, not applicable; PS-MA, poly(styrene-alt-maleic acid); PSS-MA, poly(4-styrene sulphonate-co-maleic acid); XRD, X-ray diffraction.

Strain parameters and coherence lengths derived from line profile analysis of powder synchrotron XRD spectra of calcite crystals. The numbers in brackets are the s.d. of the measurements, and the goodness of fitness of the Rietveld analysis and the *χ*^2^-distributions of the Williamson–Hall plots are listed in Supplementary Table 4.

**Table 2:** Influence of annealing and ageing on strain parameters and coherence lengths of calcite/polymer crystals.

	**Sample 1**		**Sample 2**		**Sample 3**	
	**Fresh**	***In situ*** **heating 300 **^**o**^**C**	**Fresh**	***Ex situ*** **heating 400 °C**	**Fresh**	**Aged in air 24 h**
Rietveld (pseudo-Voigt)						
Size only (nm)	224 (19)	223 (9)	388 (32)	295 (54)	300 (11)	322 (31)
Strain only (%)	0.046 (5)	0.050 (2)	0.029 (3)	0.040 (3)	0.034 (2)	0.032 (1)
Size (nm) and strain (%)	446 (23)/0.041 (5)	519 (48)/0.046 (1)	708 (55)/0.025 (2)	1,493 (123)/0.039 (7)	643 (35)/0.030 (2)	666 (43)/0.029 (2)
Williamson–Hall plot						
Size only (nm)	83 (5)	74 (4)	141 (7)	102 (5)	112 (5)	114 (6)
Strain only (%)	0.099 (3)	0.109 (3)	0.057 (1)	0.079 (1)	0.072 (1)	0.070 (2)
Size (nm) and strain (%)	2,344 (1159)/0.10 (1)	−1,812 (3087)/0.11 (1)	1,335 (609)/0.051 (5)	−1,601 (9513)/0.084 (4)	1,011 (418)/0.064 (5)	1,036 (371)/0.063 (6)
Scherrer equation (104)						
Size only (nm)	225 (12)	223 (19)	368 (45)	299 (31)	319 (27)	319 (24)
Scherrer equation (006)						
Size only (nm)	192 (21)	189 (13)	319 (32)	258 (18)	266 (14)	266 (19)

NA, not applicable; PS-MA, poly(styrene-alt-maleic acid); PSS-MA, poly(4-styrene sulphonate-co-maleic acid); XRD, X-ray diffraction.

Strain parameters and coherence lengths derived from line profile analysis of powder synchrotron XRD spectra of three different batches of calcite/PSS-MA crystals precipitated from [Ca^2+^]=5 mM and PSS-MA=125 μg ml^−1^ solution after *in situ* heating to 300 °C, *ex situ* heating to 400 °C and ageing in air. The numbers on brackets are the s.d. of the measurements, and the goodness of fitness of the Rietveld analysis and the *χ*^2^-distributions of the Williamson–Hall plots are listed in Supplementary Table 4.
