# Supplementary material for: A critical analysis of calcium carbonate mesocrystals
Source: Nat Commun. 2014 Jul 11;5:4341. doi: 10.1038/ncomms5341 (PMC4104461; doi:10.1038/ncomms5341)
Supplement: Supplementary Information — Supplementary Figures 1-9, Supplementary Tables 1-4, Supplementary Notes 1-2 and Supplementary References [file ncomms5341-s1.pdf]

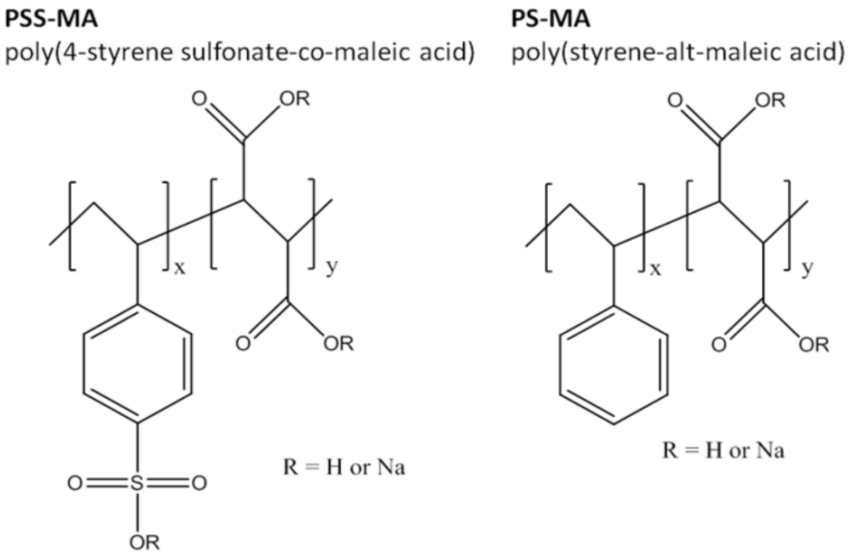

Supplementary Figure 1. Structures of the copolymers used in the experimental work.

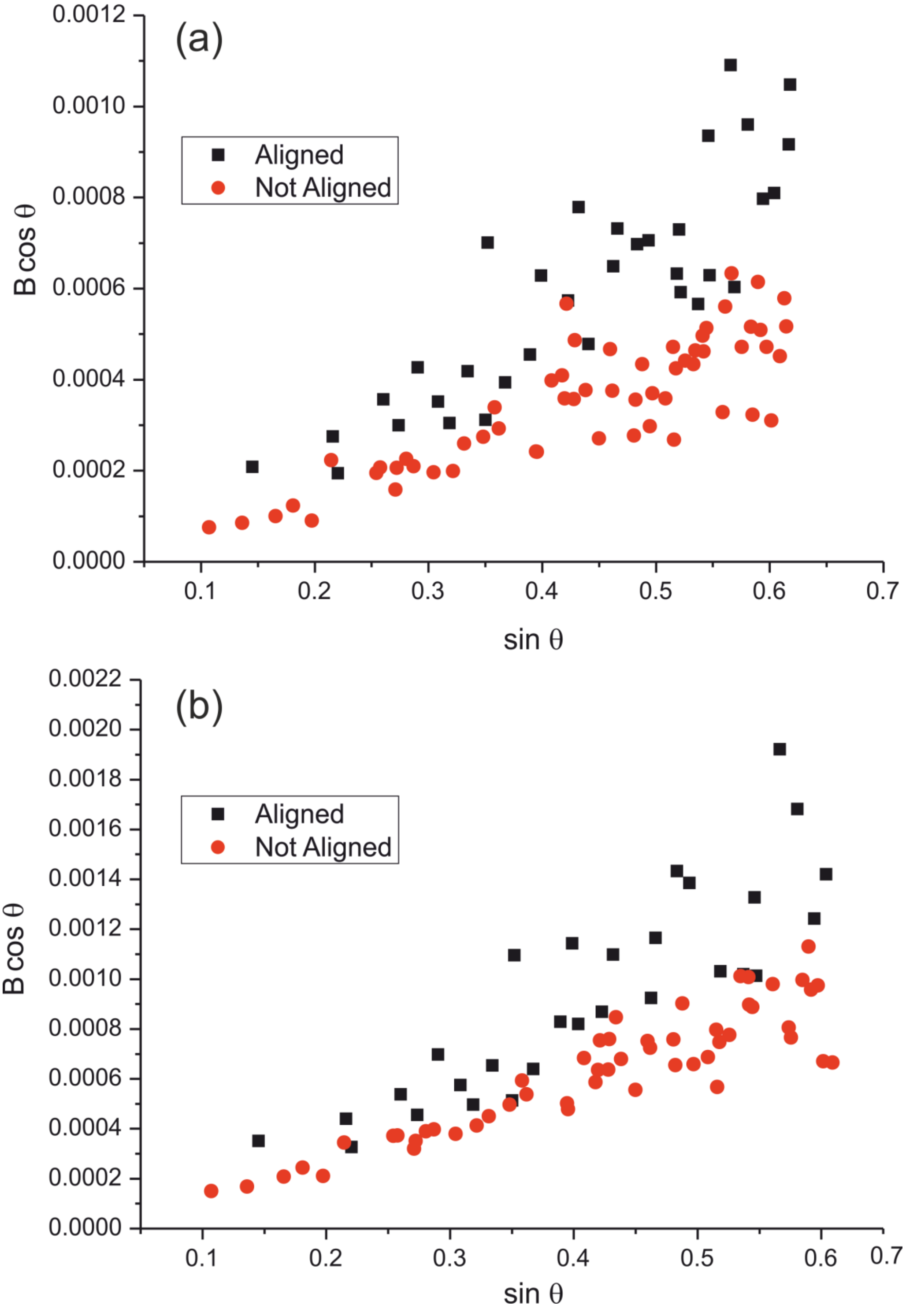

Supplementary Figure 2. Williamson-Hall plots derived from analysis of the XRD data (a) calcite/PSS-MA crystals and (b) seeded calcite/PSS-MA crystals. Highlighted are the reflections which are strongly aligned to the 001 direction (within 4 degrees, black squares) and which are less strongly aligned (red circles). θ is the diffraction angle and B is the FWHM (full-width-maximum-height) of the peak.

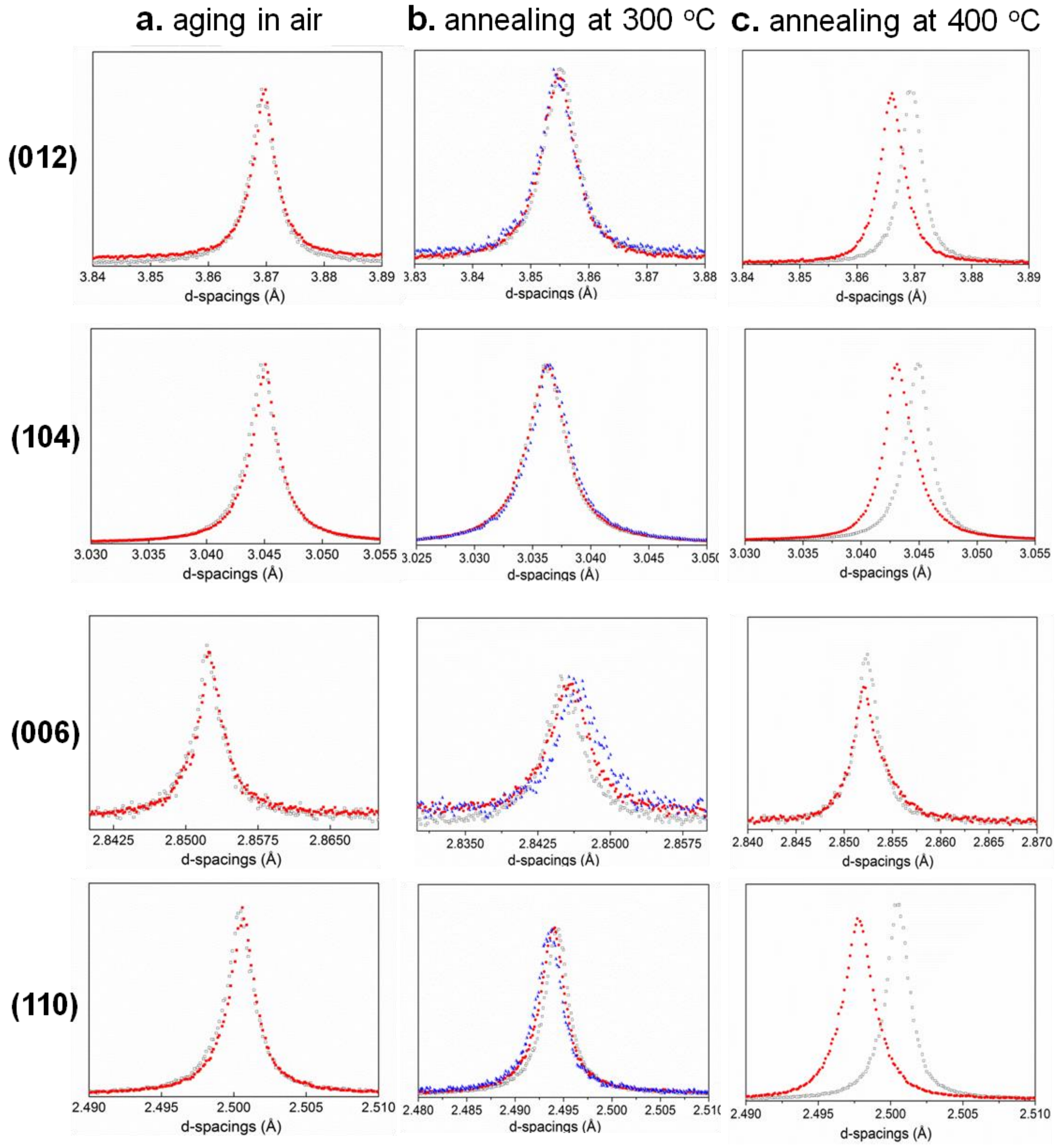

Supplementary Figure 3. Changes in XRD spectra of Calcite/PSS-MA samples after ageing and annealing. (a) XRD reflections of fresh sample (black) and after ageing in air for 24 hrs (red) and (b) XRD reflections recorded with *in situ* annealing up to 300 °C, where these show no change in intensity or broadening, and (c) XRD reflections before (black) and after (red) annealing the sample at 400 °C for 4.5 hrs).

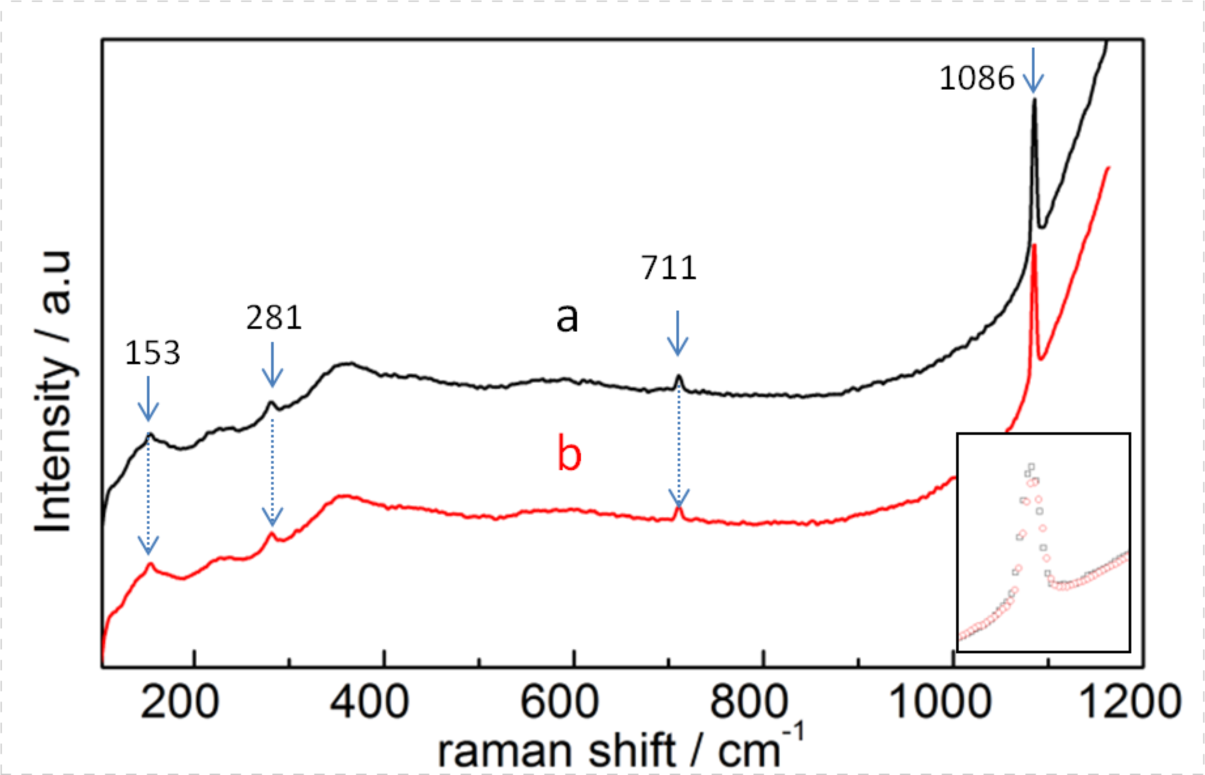

Supplementary Figure 4. Raman spectra of calcite/ PSS-MA crystals (a) immediately after isolating the crystals from solution and (b) after allowing them to age in air for 1 day. The spectra show a sharp peak at 1086 cm^{-1} (v_1) and one at 711 cm^{-1} (v_4), where these correspond to the internal CO_3^{2-} symmetric stretch and CO_3^{2-} symmetric bending respectively. The lattice mode peaks appear at 281 cm^{-1} and 153 cm^{-1}. The inset shows a magnification of the 1086 cm^{-1} (v_1) peaks.

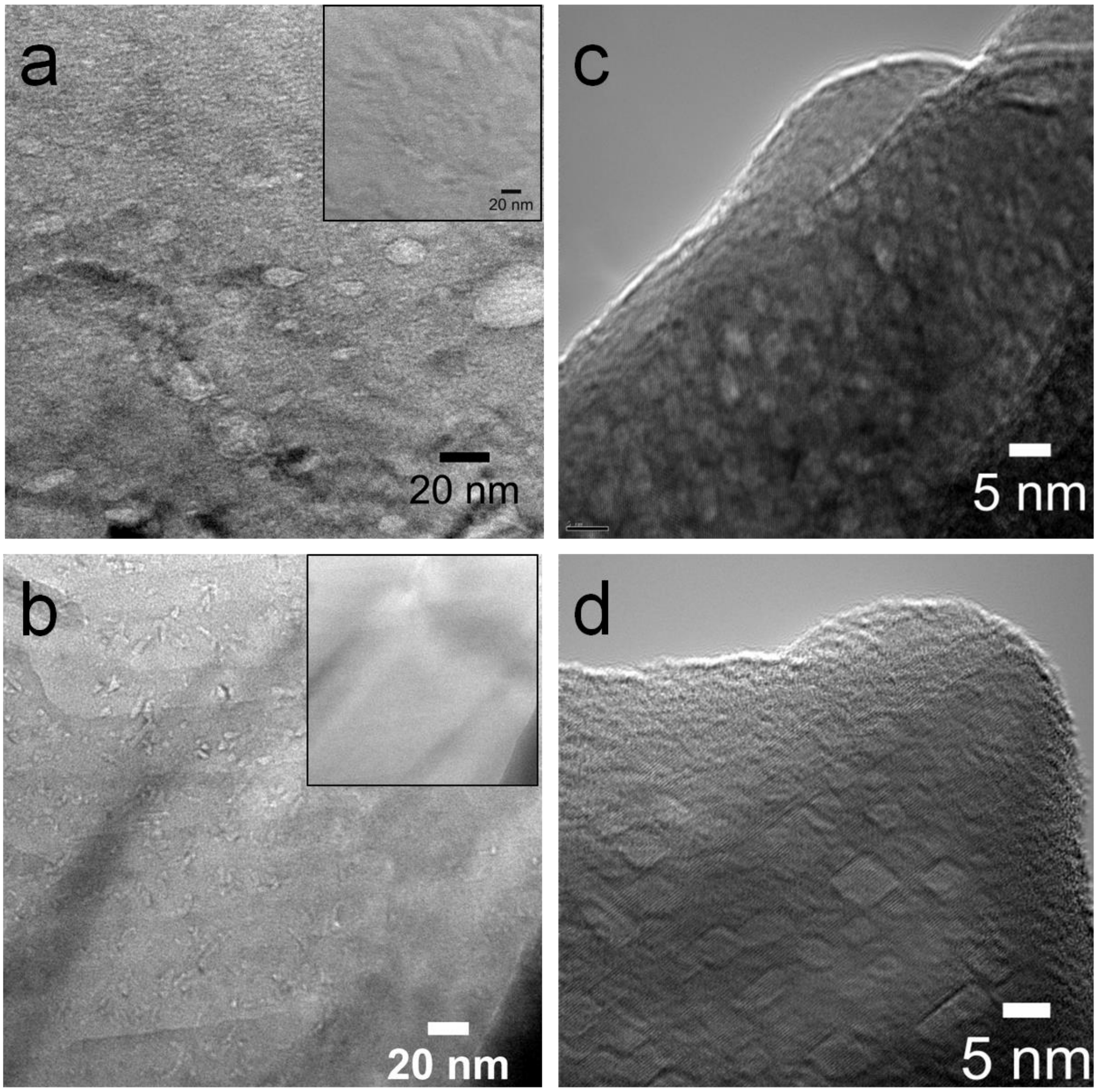

Supplementary Figure 5. **TEM images showing pore formation in calcite crystals due to electron beam irradiation**. (a) A calcite crystal precipitated in the presence of the soluble block copolymer PEG-Pasp (Poly(ethylene glycol)-Poly(aspartic acid)) showing the formation of pores after irradiation (inset: before irradiation/damage), (b) geological calcite showing much reduced beam-damage (inset : before irradiation/damage) and (c) and (d) calcite formed in the presence of 10 mg mL^{-1} of the soluble polymer, poly(acrylic acid), showing beam damage after irradiation.

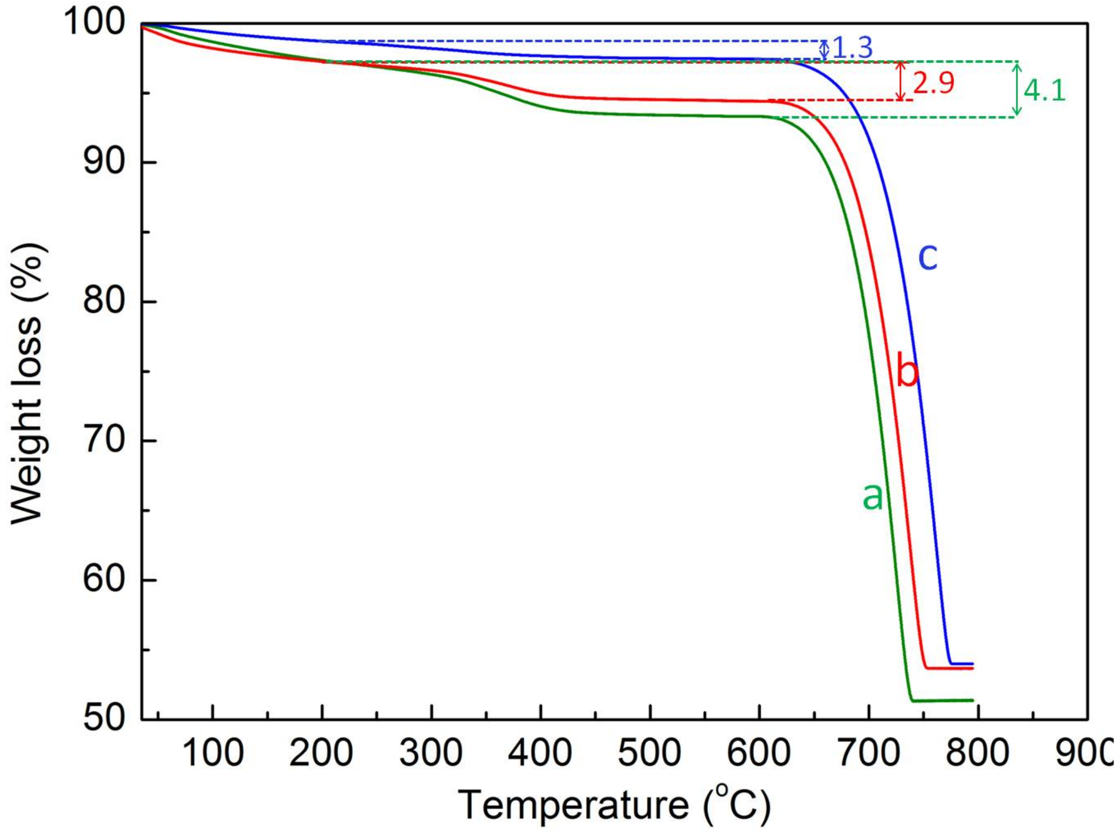

Supplementary Figure 6. TGA analysis of calcite crystals precipitated in the presence of PSS-MA from solutions for different reaction times (a and b) (a) $[Ca^{2+}]$ = 5 mM and [PSS-MA] = 125 μg ml^{-1} for 1 day showing a weight loss 4.1 wt % in the temperature range range between 200 °C and 450 °C (b) $[Ca^{2+}]$ = 5 mM and [PSS-MA] = 125 μg ml^{-1} for 10 days, showing polymer weight loss of 2.9 wt % in the range of 200 °C and 450 °C (c) crystals precipitated in solution which is undersaturated with respect to ACC. $[Ca^{2+}]$ = 0.5 mM, $[CO_3^{2-}]$ = 10 mM and [PSS-MA] = 100 μg ml^{-1} using the metastable solution method, which leads to a weight loss 1.3 wt% in the range of 200 °C and 450 °C. (The weight losses up to 200 °C were assigned to dehydration of external water, which were (a) 3.3 wt %, (b) 3 wt % and (c) 2.2 wt %.

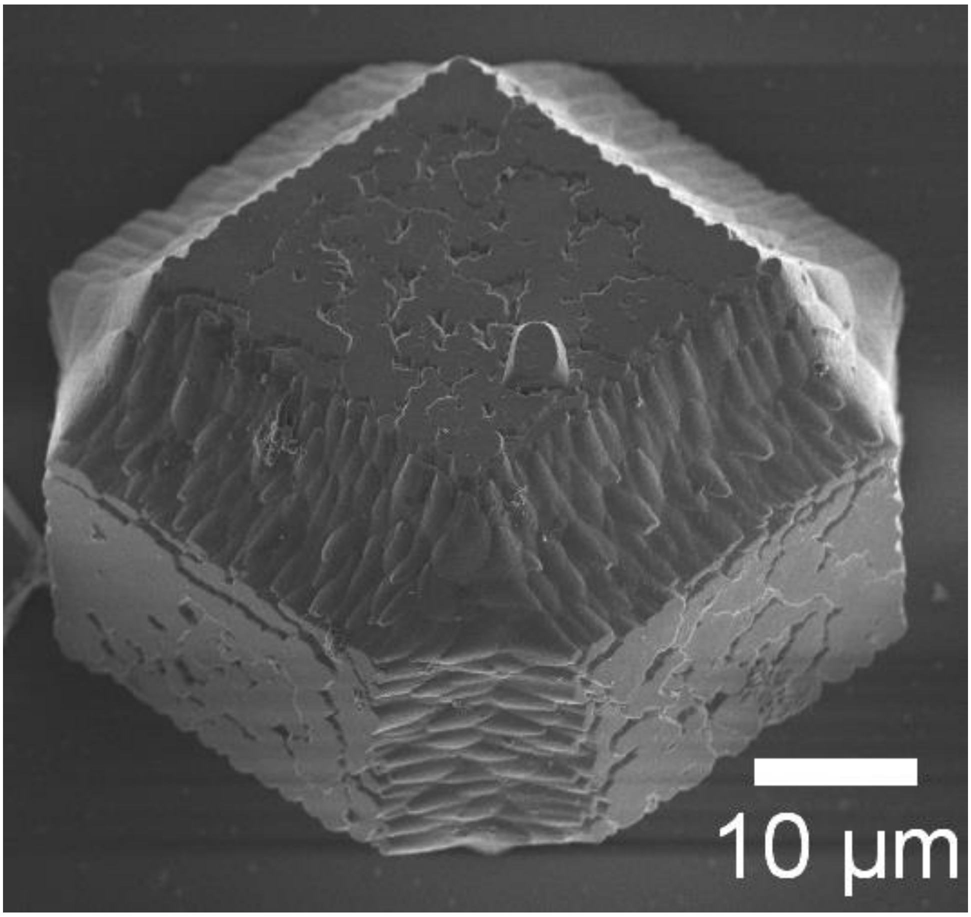

Supplementary Figure 7. **Overgrowth of large (≈ 25 µm) calcite seed crystals in a polymer-containing solution using the ammonia diffusion method**. Growth from a solution of [Ca^{2+}] = 2.5 mM and [PSS-MA] = 125 µg mL^{-1}.

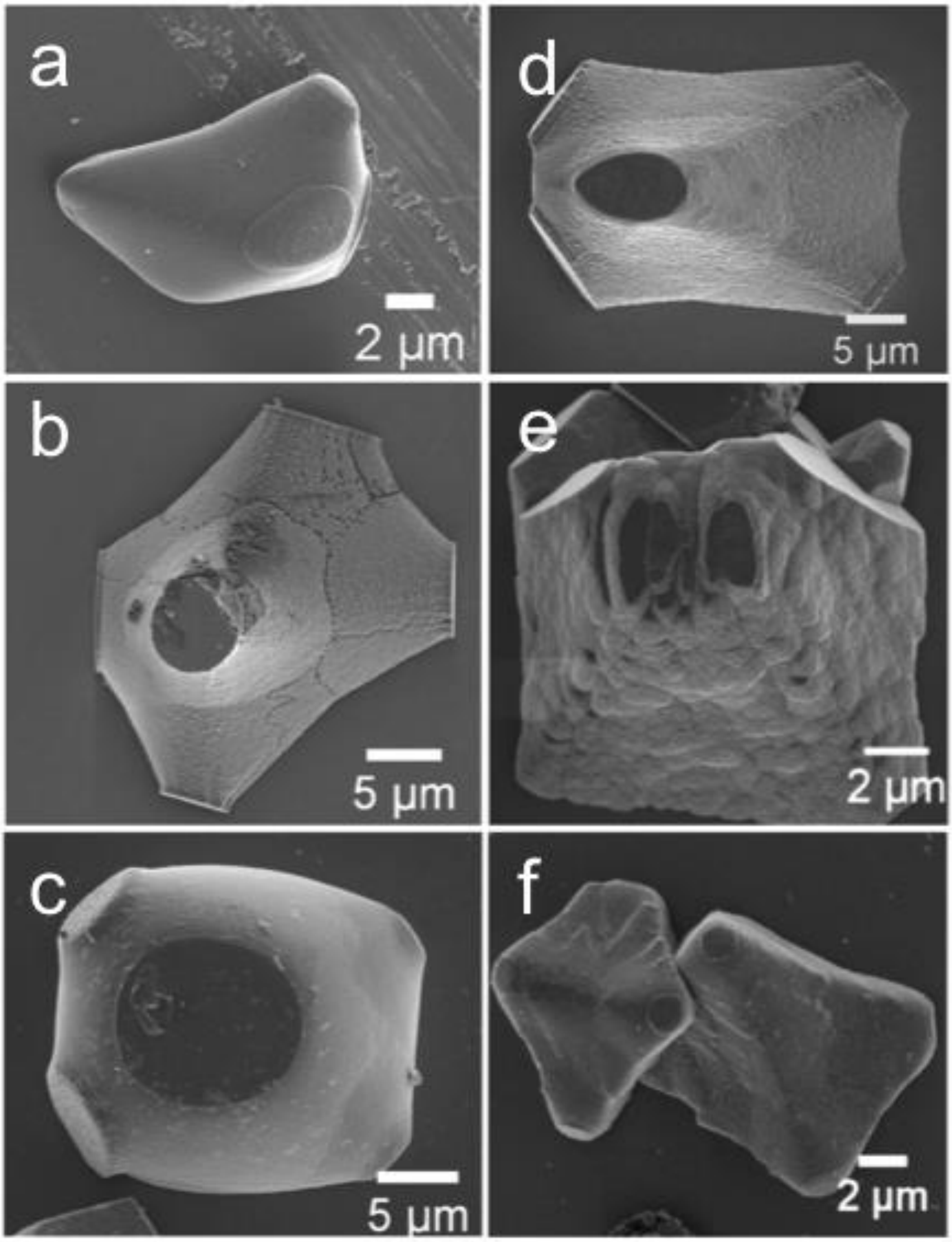

Supplementary Figure 8. Calcite crystals grown in the presence of PSS-MA from solutions which are undersaturated with respect to ACC. (a) $[Ca^{2+}]$ = 0.5 mM, $[CO_3^{2-}]$ = 5 mM and [PSS-MA] = 50 µg ml^{-1}, **$SI_{calcite}$ = 3.11 and S_{acc} = -1.67,** (b) $[Ca^{2+}]$ = 1 mM, $[CO_3^{2-}]$ = 5 mM and [PSS-MA] = 50 µg ml^{-1}, **$SI_{calcite}$ = 3.73 and S_{acc} = -1.07,** (c) $[Ca^{2+}]$ = 2 mM, $[CO_3^{2-}]$ = 5 mM and [PSS-MA] = 50 µg ml^{-1} **$SI_{calcite}$ = 4.31 and SI_{acc} = -0.45** (d) $[Ca^{2+}]$ = 0.5 mM, $[CO_3^{2-}]$ = 10 mM and [PSS-MA] = 50 µg ml^{-1} **$SI_{calcite}$ = 3.49 and SI_{acc} = -1.32** (e) $[Ca^{2+}]$ = 0.5 mM, $[CO_3^{2-}]$ = infinite and [PSS-MA] = 50 µg ml^{-1} **$SI_{calcite}$ > 4.70 and SI_{acc} > -0.19** (f) $[Ca^{2+}]$ = 2 mM, $[CO_3^{2-}]$ = 5 mM and [PSS-MA] = 200 µg ml^{-1} **$SI_{calcite}$ = 4.31 and SI_{acc} = -0.45**

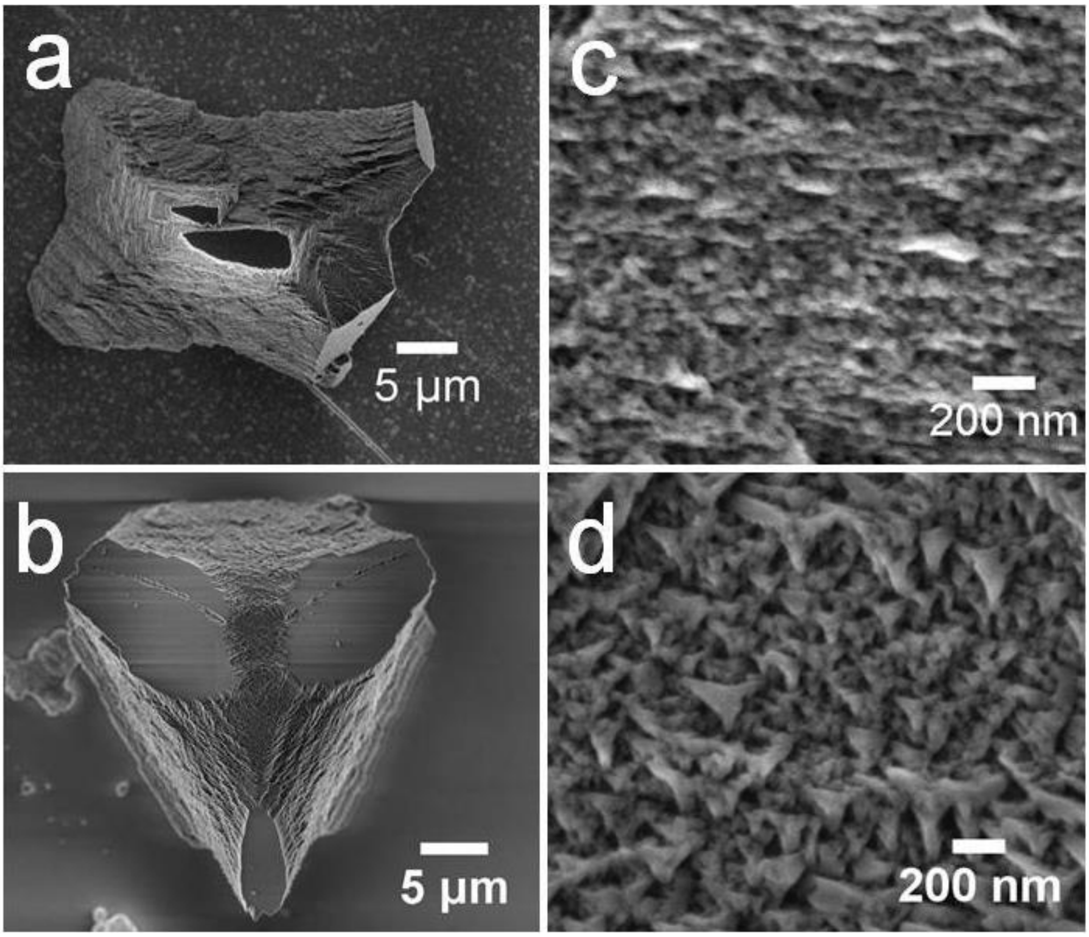

Supplementary Figure 9. SEM images of calcite crystals precipitated in the presence of PSS-MA from solutions which are undersaturated with respect to ACC. (a and c) $[Ca^{2+}]$ = 0.25 mM, $[CO_3^{2-}]$ = 100 mM and [PSS-MA] = 100 µg ml^{-1} using the metastable solution method and (b and d) $[Ca^{2+}]$ = 0.25 mM, $[CO_3^{2-}]$ = infinite and [PSS-MA] = 50 µg ml^{-1} using the ammonia diffusion method.

		Calcite Control 10 mM	PSS-MA	PSS-MA Seeded	PS-MA
**Rietveld (pseudo-voigt)**	Size only (nm)	3.69	5.37	4.003	3.15
	Strain only (%)	4.83	3.778	2.312	2.76
	Size (nm) and strain (%)	3.64	3.63	2.312	2.44
**Williamson-Hall plot**	Size only (nm)	0.00017	0.0012	0.0035	0.00040
	Strain only (%)	0.00016	0.00049	0.000798	0.00012
	Size (nm) and strain (%)	0.00013	0.00042	0.000798	0.00005

		Sample 1		Sample 2		Sample 3	
		Fresh	In situ heating 300 ^{o}C	Fresh	Ex situ heating 400 ^{o}C	Fresh	Aged in air 24hrs
**Goodness of fit (Rietveld (pseudo-voigt)**	Size only (nm)	4.50	1.68	3.46	5.10	2.65	3.40
	Strain only (%)	4.24	1.50	2.99	4.56	2.25	2.76
	Size (nm) and strain (%)	4.06	1.47	2.69	3.96	2.09	2.74
**Chi squares distribution**	Size only (nm)	0.00449	0.0064	0.00187	0.00441	0.00283	0.0026
	Strain only (%)	0.00100	0.0008	0.00033	0.00026	0.00046	0.0005
	Size (nm) and strain (%)	0.00095	0.0008	0.00031	0.00025	0.00042	0.0006

Supplementary Table 1. Goodness of fit of Rietveld analysis and Chi squares distribution of Williamson-Hall plots of data given in Table 1 and Table 2.

Reaction Conditions	Sample description	Specific surface area ($m^2 g^{-1}$)
Kitano method with Co(II) ions.[1]	Co-calcite	0.47
Biomineral	Ground Sea urchin plate	1.2
[Ca^{2+}1 =10 mM, ADM	Synthetic calcite 20- 50 µm	0.15
Commercial	Nano calcite, 50-100 nm	22
**Calcite/PSS-MA crystals**		
**Influence of Ageing Dry Samples in Air**		
[Ca^{2+}] = 5 mM<br>[PSS-MA]= 125 µg/mL	**Not aged**	57.03
[Ca^{2+}] = 5 mM<br>[PSS-MA]= 125 µg/mL	**Aged for 24 hrs**	25.78
[Ca^{2+}] = 5mM<br>[PSS-MA]= 125 µg/mL	**Aged for 2 months**	4.59
**Sample Prepared by Overgrowth of Seed Crystal**		
[Ca^{2+}] = 2.5 mM<br>[PSS-MA] = 125 µg/mL	Not Aged	27.64
[Ca^{2+}] = 2.5 mM<br>[PSS-MA] = 125 µg/mL	Aged for 14 days	5
**Crystals grown at Low Supersaturations**		
[Ca^{2+}] =0.5 mM, **[CO_3^{2-}] = 10 mM**<br>[PSS-MA] = 100 µg/mL	Low Supersaturation<br>**SI_{ACC} = -1.375**	1.93
[Ca^{2+}] = 0.5 mM **[CO_3^{2-}] = 100 mM**<br>[PSS-MA] = 100 µg/mL	Low Supersaturation<br>**S_{ACC} = -0.744**	4.80
**Crystals grown in the Presence of Different Polymer Concentrations**		
[Ca^{2+}] = 5 mM [CO_3^{2-}] = 100 mM<br>**[PSS-MA] = 300 µg/mL**	High polymer concentration	55.93
[Ca^{2+}] = 5 mM [CO_3^{2-}] = 100 mM<br>**[PSS-MA] = 150 µg/mL**	low polymer concentration	40

Supplementary Table 2. Specific surface areas of calcite reference samples and particles precipitated in the presence of polymer additives and Co(II) ions, as measured using BET. Crystals were precipitated using the ammonia diffusion method unless stated.

Mass of Powder Analysed	1003 mg	125 mg	15 mg	10 mg
Surface area (m^2 g^{-1})	21.2095 +/- 0.1070	16.6741 +/- 0.4975	11.4877 +/- 1.8591	3.0658 +/- 1.0140
Correlation coefficient	0.9999630	0.9987406	0.9678721	0.9196139

Supplementary Table 3: **Dependence of surface areas of samples on the mass of sample analysed**. Commercial nanocalcite, with sizes of 50-100 nm was investigated, where data was analysed using the BET method based on 5 point surface area measurements.

Added $[Ca^{2+}]$ and [PSS-MA]	Measured $[Ca^{2+}]$ (mM)	Added $[CO_3]$ (mM)	Supersaturation (calcite) = ln (IAP/Ksp) calcite	Super/Under-saturation (ACC) =Ln (IAP/Ksp) ACC
$[Ca^{2+}]$ = 0.5 mM [PSS-MA] = 50 µg mL^{-1}	$[Ca^{2+}]$ = 0.471 mM	5	3.05	-1.76
		10	3.43	-1.38
		100	4.06	-0.74
		400	4.30	-0.51
$[Ca^{2+}]$ = 1mM [PSS-MA] = 50 µg mL	$[Ca^{2+}]$ = 0.96 mM	5	3.70	-1.11
$[Ca^{2+}]$ = 2 mM [PSS-MA] = 50 µg mL	$[Ca^{2+}]$ = 1.878 mM	5	4.26	-0.55
$[Ca^{2+}]$ = 5 mM [PSS-MA] = 200 µg mL^{-1}	$[Ca^{2+}]$ = 4.75 mM	5 - 100	4.91 - 6.35	0.10 – 1.54

Supplementary Table 4: Summary of the supersaturation index of selected reaction solutions.

Supplementary Note 1

XRD Analysis. X-ray diffraction peak broadening is caused by contributions from the instrument, the crystallite domain size and lattice distortion caused by stress or strain. For our samples this was modelled using the Scherrer equation, a Williamson-Hall plot and Rietveld refinement, where the results are presented in Tables 1 and 2. Application of the Scherrer equation involves taking the peak full width half maximum (FWHM), removing the instrumental broadening by comparison with the FWHM of a highly crystalline sample, and then assuming that all remaining broadening is caused by particle size effects alone. This has the virtue of being a trivial method of providing quantitative data on particle size for comparison between samples where this is likely to be the major contributor to peak broadening (eg. nanoparticles). However, in most cases ignoring stress-strain effects is far too simplistic a model.

In common with Scherrer, the Williamson-Hall technique makes use of adjusted FWHM values, but this time separates out size and strain effects on peak broadening based on how they vary with the diffraction angle, θ, with size broadening being proportional to sec θ, and strain to tan θ. In contrast to Rietveld refinement, the whole peak shape (including the instrumental component) is modelled using a pseudo-voigt function using at least 6 parameters to define the variation in shape as a function of the diffraction angle. The model is refined to fit the whole pattern and provides a much more sophisticated method of describing the instrumental broadening. This explains the difference in the data derived using the Rietveld and Williamson-Hall methods (Tables 1 and 2), where both validate each other by showing the same trends.

Supplementary Note 2

Small Angle X-Ray Scattering (SAXS). Analysis of the diffuse elastic scattering around the primary beam (origin of reciprocal space) provides information about size and shape of structural motifs generally in the size range between 1 nm and 100 nm. As an advantage of this method, structural information can be extracted with a high statistical accuracy, since the entire sample volume illuminated by the x-ray beam is considered for the evaluation. However, SAXS is an indirect method, deriving information from reciprocal space such that appropriate models are required to derive structural parameters from the data.[7]

Cobalt-ion doped calcite gave rise to a scattering profile showing little structural complexity as manifested in a curve shape similar to that obtained from a control calcite sample (Main Paper,

Figure 3). The latter shows a Porod-like ($I(Q) \propto Q^{-4}$) behaviour over the entire range of the data set. Since the SAXS intensity can be described by the Porod law,[8] only in two-phase systems with well-defined sharp interfaces, where this represents the tail of the radially averaged intensity profile (i.e. $R \cdot Q >> 1$, with R denoting the size of the scattering objects), we can conclude that the scattering of the pure calcite crystals is dominated by the smooth micrometer-sized external facets of the powder grains. There is no indication of nanoscale internal structural features. The profile recorded for the specimen accommodating foreign ions, however, deviates from this behaviour at small values of the modulus of the scattering vector Q, where the scattering intensity originating from larger structures (> 30 nm) is represented. This deviation might be caused by roughness on the external facets of the Co-doped calcite or electron density heterogeneities on a length scale larger than what was previously observed for porous calcite/poly(styrene sulphonate) (PSS) crystals.[9] The nanostructures of seeded calcite/ PSS-MA crystals and the calcite/PS-MA crystals were then compared. The radially averaged intensity profiles obtained from these specimens are shown in Figure 3. In common with the profiles of calcite-PSS particles previously studied by Schenk et al.,[9] the curves can be divided into three regimes. The scattering at low Q (regime 1) is dominated by a steep linear decay, which presumably originates from the large external facets of the powder grains, whereas smaller nanostructural features within the mineral particles give rise to a bent curve shape in regime 2. In the limit of high Q values (regime 3) the profile shows a linear decay related to the structure of the internal interfaces (here assumed to be Porod-like, i.e. smooth).

An evaluation of the Guinier region in the SAXS profile generated by calcite/PS-MA crystals ($0.25\ nm^{-1} < Q < 1.3\ nm^{-1}$, regime 2) based on the modified Guinier approximation $I(Q) \propto Q^{-2}\exp(-Q^2D^2/12)$[7] points to dilute platelet-shaped occlusions/pores within these crystals, where a value of D = 2.9 nm was determined for the average thickness of the platelets. Guinier functions valid for dilute spherical or cylindrical objects, in contrast, did not provide a satisfactory fit for the data.

In order to further estimate the mean size of the scattering objects present within the calcite/PSS-MA and calcite/PS-MA samples considering the scattering intensity at larger values of Q (corresponding to smaller structures), a T-parameter analysis (assuming a 2-phase system composed of a calcite matrix with occlusions of polymer) was additionally performed. The T-parameters were calculated according to the equation $T = \frac{4 \cdot \tilde{I}}{\pi \cdot P} = \frac{4\phi \cdot (1-\phi)}{S}$ and can be understood as a measure for the volume fraction ϕ (proportional to the integral intensity $\tilde{I} = \int_0^\infty I(Q) \cdot Q^2 dQ$) per specific surface area S (proportional to the Porod constant P) of one of the two phases present within the mineral/polymer crystals. The methodology for the determination of the T-parameter has

previously been applied to the mineral crystals in bone and was described in detail by Fratzl et al. [6] In the case of dilute platelet-shaped heterogeneities within a sample, the T-parameter can be associated with 2·D, D being the thickness of the platelets. [10] From the T-parameters obtained in our experiments (T = 3.8 nm for calcite/PSS-MA and T = 3.4 nm for calcite/PS-MA), values of D = 1.9 nm and D = 1.7 nm were determined for the thickness of the platelet-shaped occlusions within calcite crystals grown in the presence of PSS-MA and PS-MA, respectively. In the case of calcite/PS-MA, the thus obtained thickness of 1.7 nm is smaller than the one extracted from the Guinier fit. The parameters calculated from both analyses are consistent, though, since when there is a size distribution of the scattering objects (polydispersity), the T-parameter inherently emphasizes the smaller structures (with larger surface), whereas the Guinier thickness emphasizes the larger objects.

Supplementary References

1 Kitano, Y., Hood, D. W. & Park, K. Pure Aragonite Synthesis. *J Geophys Res* **67**, 4873-&, (1962).

2 Braybrook, A. L., Heywood, B. R., Jackson, R. A. & Pitt, K. Parallel computational and experimental studies of the morphological modification of calcium carbonate by cobalt. *J. Cryst. Growth* **243**, 336-344, (2002).

3 Stephens, C. J., Kim, Y.-Y., Evans, S. D., Meldrum, F. C. & Christenson, H. K. Early Stages of Crystallization of Calcium Carbonate Revealed in Picoliter Droplets. *J. Am. Chem. Soc.* **133**, 5210-5213, (2011).

4 Fitch, A. N. The high resolution powder diffraction beam line at ESRF. *J. Res. Nat. Inst. Stand. Technol.* **109**, 133-142, (2004).

5 Caglioti, G., Paoletti, A. & Ricci, F. P. Choice of Collimators for a Crystal Spectrometer for Neutron Diffraction. *Nucl Instrum Methods* **3**, 223-228, (1958).

6 Fratzl, P., Schreiber, S. & Klaushofer, K. Bone mineralization as studied by small-angle x-ray scattering. *Connec. Tiss. Res.* **34**, 247-254, (1996).

7 Glatter, O. & Kratky, O. (Academic Press, London, 1982).

8 Porod, G. Die Rontgenkleinwinkelstreuung Von Dichtgepackten Kolloiden Systemen .1. *Kolloid-Zeitschrift and Zeitschrift Fur Polymere* **124**, 83-114, (1951).

9 Schenk, A. S. *et al.* Hierarchical Calcite Crystals with Occlusions of a Simple Polyelectrolyte Mimic Complex Biomineral Structures. *Adv.Func. Mater.* **22**, 4668-4676, (2012).

10 Fratzl, P. Statistical Model of the Habit and Arrangement of Mineral Crystals in the Collagen of Bone *J. Stat. Phys.* **77**, 125-143, (1994).
